# Interpreting antimicrobial resistance from bacterial whole-genome sequencing: prediction tools, database fragmentation, analytical trade-offs, and harmonized reporting

**DOI:** 10.3389/fmicb.2026.1849165

**Published:** 2026-09-14

**Authors:** Abdullateef Abdullah Alshehri

**Affiliations:** 1 Department of Clinical Laboratory Sciences, Faculty of Applied Medical Sciences, Najran University, Najran, Saudi Arabia; 2 Department of Public Health, Infection Prevention and Control, Najran University Hospital, Najran University, Najran, Saudi Arabia; 3 Genomics and Genetics Unit, Health Research Center, Najran University, Najran, Saudi Arabia

**Keywords:** antibiotic-resistance genes (ARGs), antimicrobial resistance (AMR), antimicrobial susceptibility testing (AST), database harmonization, genotype–phenotype discordance, One Health, whole-genome sequencing (WGS)

## Abstract

Whole-genome sequencing (WGS) has become a critical component of antimicrobial resistance (AMR) surveillance because it can characterize bacterial lineages, resistance determinants, and, when sequence resolution is sufficient, the mobile genetic elements that mediate dissemination. However, the practical value of WGS-based AMR inference remains constrained by fragmentation across AMR databases, inconsistent nomenclature, variable curation practices, and differences in analytical thresholds and reporting rules. Consequently, the same isolate may yield discordant resistome outputs across tools, limiting reproducibility, cross-study comparability, and surveillance integration. This review focuses on the interpretation of bacterial WGS data for AMR detection, with emphasis on AMR prediction tools, reference databases, read-mapping and assembly-based workflows, genotype–phenotype discordance, validation strategies, and harmonized reporting. General bioinformatics steps, including quality control, assembly, and polishing, are discussed only where they directly affect AMR inference, such as small-variant detection, plasmid reconstruction, and mobile genetic element context. The review further evaluates major AMR resources with respect to scope, curation depth, evidence models, updating practices, and interoperability across clinical and One Health applications. Rather than advocating a single universal database, we argue that the field would benefit more from federated harmonization based on shared ontologies, transparent provenance, versioned crosswalks, and benchmarked reporting standards. Within this context, AMR-GenoLink is introduced as a proposed reference framework for interoperable ingestion, standardized reporting, and provenance-aware integration of WGS-derived AMR evidence across human, animal, and environmental domains. The framework separates genomic feature detection from resistance interpretation, phenotype-linked validation, and evidence-proportionate reporting. Overall, this review argues that reliable WGS-based AMR interpretation is increasingly constrained not only by limitations in resistance-gene detection but also by insufficient harmonization across databases, analytical workflows, validation standards, and reporting frameworks.

## 1 Introduction

Antimicrobial resistance (AMR) is a major global health challenge and an escalating threat to effective treatment and infection prevention. The World Health Organization (WHO), the Centers for Disease Control and Prevention (CDC), and the European Committee on Antimicrobial Susceptibility Testing (EUCAST) have emphasized the urgency of coordinated action to address AMR. Under a no-action scenario, AMR-associated mortality has been projected to reach almost 10 million annually by 2050 (World Health Organization [WHO], 2020, 2025; Giske et al., 2022; Murray et al., 2022; European Centre for Disease Prevention and Control [ECDC] et al., 2024; Naghavi et al., 2024). Key drivers include the overuse of antibiotics and the limited development of novel antimicrobial agents (Murray et al., 2022; World Health Organization [WHO], 2022; Naghavi et al., 2024). Together, these pressures challenge the effectiveness of innovation and scalable surveillance strategies.

Historically, bacterial AMR surveillance primarily depended on antimicrobial susceptibility testing (AST) and other culture-based phenotypic techniques (as illustrated in Figure 1), such as disk diffusion and minimum inhibitory concentration (MIC) determination, which support treatment decisions but provide limited insight into resistance mechanisms and transmission (Franco-Duarte et al., 2019; Arbefeville et al., 2024). Genotype-based methods such as PCR and microarrays can detect known determinants and mobilizable elements, yet their interpretation is constrained by assay scope, database content, regulation-dependent effects, and genotype–phenotype discordance (Zankari et al., 2012; Rose et al., 2023; Hassall et al., 2024). Accordingly, guidance emphasizes benchmarking genomic predictions against phenotypic AST for clinical interpretation. Importantly, detection of an antibiotic-resistance gene (ARG) or resistance-associated mutation does not by itself confirm phenotypic resistance, which can depend on genome-wide context, regulation, expression, and copy number. Genomic surveillance has accelerated this shift, enabling high-resolution characterization of bacterial evolution, transmission, and resistance mechanisms.

**FIGURE 1:**
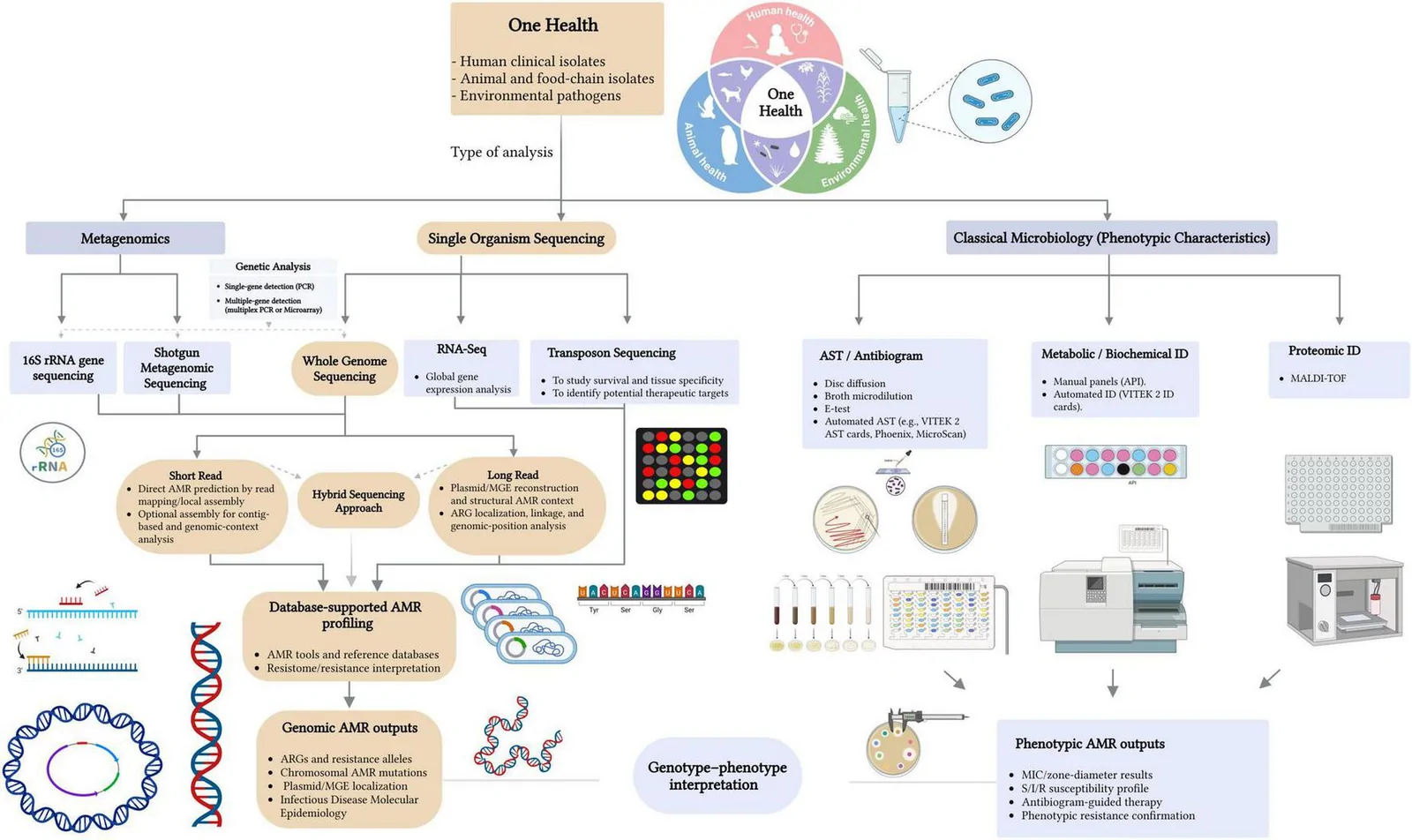
Conceptual overview of AMR interpretation across classical microbiology, isolate whole-genome sequencing (WGS), and metagenomic analysis within a One Health framework. Classical microbiology provides phenotypic antimicrobial susceptibility testing (AST) evidence, while isolate WGS enables detection of acquired resistance genes, resistance-associated mutations, plasmid/MGE context, and molecular epidemiology. WGS and, where applicable, metagenomics can support lineage typing, resistome profiling, mobilome characterization, surveillance, outbreak investigation, and contextual interpretation of AMR determinants within mobile genetic elements. Short-read data can support direct AMR detection through read mapping or local assembly, whereas *de novo* assembly is generally required for contig-based screening and genomic-context interpretation. Long-read and hybrid workflows improve plasmid and mobile genetic element resolution when structural AMR context is required. Metagenomic workflows support community-level resistome profiling in environmental, wastewater, animal, and other mixed-sample settings. The figure was created with BioRender.com (accessed 29 Aug 2026). Alshehri, A. (2026) https://BioRender.com/p57k871.

The advancement of high-throughput sequencing (HTS), particularly whole-genome sequencing (WGS), has fundamentally transformed this domain by facilitating detailed, high-resolution analysis of bacterial genomes (Metzker, 2010). WGS has become a critical component of modern clinical and public health microbiology, providing comprehensive genomic resolution that supports tracking of pathogen evolution, characterization of AMR mechanisms, and optimization of outbreak detection and response strategies. Coupled with modern bioinformatics, WGS enables analysis of large genomic datasets that can inform public health surveillance, outbreak investigation, and AMR interpretation. Despite the rapid growth of genomic data, there are still gaps in how quickly and smoothly WGS can be integrated into real-time public health practices and decision-making (Sherry et al., 2025).

Whole-genome sequencing has strengthened AMR surveillance by improving outbreak investigation, clarifying transmission pathways, and supporting the identification of resistance mechanisms. It has contributed to the resolution of hospital outbreaks involving multidrug-resistant (MDR) pathogens, such as tracing transmission and guiding infection prevention and control (David et al., 2019). Recent molecular studies have indicated that WGS can support detailed genomic profiling of highly resistant organisms (Owaid and Al-Ouqaili, 2025). Beyond cataloging genes, WGS can also capture factors shaping gene flow: CRISPR–Cas (clustered regularly interspaced short palindromic repeats–CRISPR-associated proteins) systems, which influence AMR profiles primarily indirectly by restricting horizontal gene transfer (HGT) through interference with incoming mobile genetic elements (MGEs), such as plasmids and integrative conjugative elements. This mechanism reduces the acquisition of horizontally transferred resistance genes (Garneau et al., 2010; Wheatley and MacLean, 2021). In some contexts, CRISPR spacers can target resistance-bearing plasmids, thereby diminishing their establishment or stability (Jwair et al., 2023; Al-Ouqaili et al., 2025; Owaid and Al-Ouqaili, 2025). In parallel, national surveillance programs increasingly employ WGS to support real-time monitoring, enabling earlier detection of resistance gene dissemination and more coordinated public health responses (Ellington et al., 2017). Nevertheless, although several promising WGS-based AMR monitoring models have been proposed, persistent challenges continue to limit their routine, scalable, and efficient implementation across healthcare and surveillance environments (Kwong et al., 2015).

Despite its substantial promise, WGS implementation for AMR surveillance remains highly variable across laboratories and regions. This variation reflects differences in policy and funding priorities, deficiencies in infrastructure and supply chains, a limited workforce in genomics and bioinformatics, non-standardized quality control (QC), validation, reporting practices (including alignment with EUCAST/CLSI), fragmented data governance, and unresolved trade-offs between cost and turnaround time when compared to traditional AST (Quainoo et al., 2017; Bonvegna et al., 2022; Papp and Solymosi, 2022; Purushothaman et al., 2022; Simner et al., 2022; Price et al., 2023; Djordjevic et al., 2024). The complexity of bacterial AMR underscores the need for integrated genomic approaches capable of capturing multiple resistance mechanisms within the same isolate. This phenomenon is exemplified by highly resistant *Klebsiella pneumoniae*, in which efflux-associated components such as AcrA and TolC may coexist with β-lactamase determinants, including extended-spectrum β-lactamases and metallo-β-lactamases (ESBLs/MBLs). Such multilayered resistance profiles highlight the importance of comprehensive genomic tools for simultaneous identification of co-occurring resistance mechanisms in clinically significant pathogens (Hussein et al., 2024). Although several studies have proposed models to improve particular aspects of AMR monitoring (Lee et al., 2021; Meroni et al., 2022; Perestrelo et al., 2023), insufficient surveillance coverage and methodological heterogeneity continue to hinder global comparability and emphasize the need for standardized workflows and mutually accepted reporting practices.

WGS-based AMR inference typically combines two complementary analytical layers: detection of acquired resistance genes and identification of chromosomal mutations affecting drug targets or regulatory pathways. However, surveillance conclusions can differ depending on how resistance determinants are contextualized within plasmids and other MGEs that drive dissemination, persistence, and co-selection. Moreover, AMR resources differ in scope, nomenclature, evidence grading, update frequency, and interpretation logic, meaning that results are not always directly comparable unless database versions, analytical thresholds, and reporting conventions are explicitly documented. These constraints strengthen the case for interoperable, versioned AMR resources and reproducible pipelines that connect resistome calls to mobilome context in a way that supports reproducible surveillance.

The One Health framework, which integrates human, animal, and environmental sectors, is essential for the surveillance of AMR and the execution of coordinated response strategies. To support this goal, AMR databases and surveillance systems should enable cross-sector data integration through standardized metadata, controlled vocabularies, and harmonized reporting structures that improve comparability across domains (Queenan et al., 2016; Kahn, 2017; World Health Organization [WHO], 2020, 2022). Within this context, AMR-GenoLink is presented as a proposed conceptual reference architecture for provenance-aware integration of isolate-level WGS outputs with population-level insights and harmonized reporting across One Health settings. It emphasizes interoperable data ingestion, reproducible analysis, and transparent translation of genomic outputs into surveillance- and stewardship-relevant reporting.

In this review, ARG detection refers to the identification of acquired resistance genes or gene variants from sequence data; resistance inference refers to the interpretation of genes, alleles, chromosomal mutations, and genomic context as evidence for potential resistance; resistome analysis refers to the broader characterization of resistance-associated determinants within an isolate, population, or metagenomic sample; and mobilome context refers to the association of AMR determinants with plasmids, integrons, insertion sequences, transposons, or other MGEs. These distinctions are important because AMR prediction tools differ in whether they detect acquired genes, curated chromosomal mutations, plasmid replicons, metagenomic ARG profiles, or broader resistance-associated mechanisms. Therefore, WGS-based AMR analysis is treated as a four-step evidence pathway. Detection identifies genes, alleles, mutations, plasmid replicons, or mobile genetic element markers. Interpretation assesses their likely resistance relevance in a defined organism–drug context. Validation compares that inference with phenotypic AST, MIC data, ECOFFs, clinical breakpoints, or other orthogonal evidence. Reporting communicates the finding according to its evidence strength and intended use. These stages should not be conflated, because detection of a genomic determinant alone does not establish phenotypic resistance or clinical actionability.

Accordingly, this review focuses on WGS-based AMR interpretation rather than general bacterial genome analysis. It critically examines how AMR prediction tools, AMR reference databases, read-mapping and assembly-based workflows, genotype–phenotype discordance, and database fragmentation shape the reliability of AMR inference. General bioinformatics processes, including QC, assembly, polishing, phylogenetic analysis, and genome annotation, are discussed only where they directly influence resistance detection, plasmid or MGE context, small-variant interpretation, or surveillance reporting. The review further evaluates why harmonized databases, expert curation, validated thresholds, and phenotypic AST benchmarking remain essential for clinical and applied interpretation. Finally, AMR-GenoLink is presented as a proposed conceptual framework for evidence-tiered, provenance-aware integration of WGS-derived AMR outputs across One Health surveillance settings.

## 2 Sequencing strategies for WGS-based AMR inference

WGS has contributed to a transition in AMR surveillance from phenotype-based summaries toward genomic-resolution analysis, enabling SNP-level transmission inference and resistance interpretation in its genetic context, including gene, allele, and associated MGE information (Goodwin et al., 2016; Ferdinand et al., 2021; Struelens et al., 2024). However, the public health value of WGS depends on matching the sequencing strategy to the primary surveillance objective. Some applications prioritize high-confidence SNP phylogenetics for outbreak detection and transmission analysis, whereas others require reconstruction of plasmids, transposons, integrons, and related MGEs to understand how resistance disseminates. Routine implementation additionally depends on scalability, cost-efficiency, and reproducibility across laboratories. Current evidence, therefore, supports a complementary rather than competitive view of sequencing platforms: short-read sequencing remains well-suited to variant-centric surveillance, whereas long-read and hybrid strategies are particularly advantageous when full genomic context is needed to interpret AMR dissemination. When appropriately deployed and validated, platform-specific workflows can support surveillance outputs for outbreak response, transmission analysis, and AMR interpretation (Ellington et al., 2017; Armstrong et al., 2019; Ferdinand et al., 2021; Bogaerts et al., 2024; Dabernig-Heinz et al., 2024; Sanderson et al., 2024; Sherry et al., 2025). An overview is given in Figure 2 and Table 1.

**FIGURE 2:**
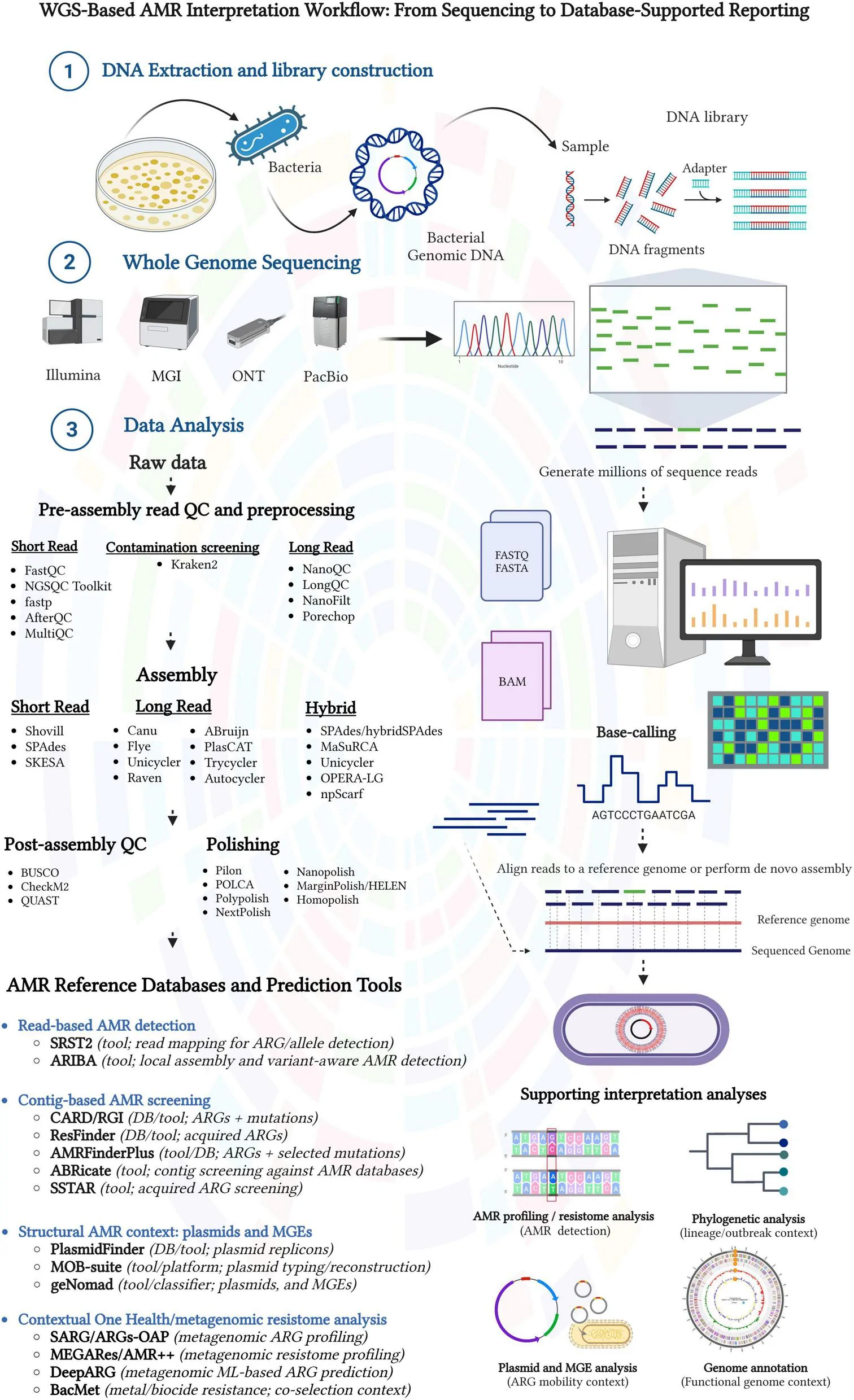
Antimicrobial resistance (AMR)-focused bacterial whole-genome sequencing analysis workflow. The pipeline depicts how short-read, long-read, and hybrid sequencing data can each support AMR analysis depending on the surveillance objective. Short-read workflows are commonly used for read-mapping or assembly-based detection of acquired antibiotic-resistance genes (ARGs) and chromosomal mutations, whereas long-read and hybrid workflows are particularly useful when plasmid reconstruction, MGE localization, or structural context is required. Hybrid sequencing is not a prerequisite for AMR analysis, but can improve interpretation when both base-level accuracy and genomic context are needed. Representative examples are shown; detailed general-purpose tool lists are in the Supplementary Tables 1–9. The figure was created with BioRender.com (accessed 29 Aug 2026). Alshehri, A. (2026) https://BioRender.com/ny5l2kz.

**TABLE 1:** Sequencing strategies and their AMR-relevant strengths and limitations in bacterial WGS workflows.

Platform	Key AMR-relevant strengths	Read length	Main errors	AMR-relevant limitation	References
Illumina	High base accuracy, scalable throughput, and relatively low per-sample cost; well-suited for SNP-based surveillance, detection of many acquired ARGs, and routine isolate screening	2 × 50 to 2 × 300 bp, depending on platform, kit, and run configuration	Low-frequency substitution errors	Limited read length can fragment assemblies and reduce the resolution of plasmids, repeat-rich regions, transposons, and other MGEs	Bentley et al., 2008; Jeon et al., 2021
PacBio (HiFi)	Long HiFi reads with high consensus accuracy can support more contiguous assemblies, plasmid reconstruction, insertion-sequence resolution, and near-complete or closed bacterial genomes	Commonly ∼10–25 kb	Earlier CLR reads had higher random error rates; HiFi reads reduce this limitation through circular consensus sequencing	Higher per-sample cost and workflow requirements compared with routine short-read sequencing; interpretation still depends on depth, library quality, and assembly validation	Wenger et al., 2019; Nurk et al., 2020; Luan et al., 2024
MGI-DNBSEQ	High-throughput short-read sequencing with low index misassignment; suitable for large-scale bacterial WGS and routine surveillance workflows	Commonly 2 × 150 to 2 × 300 bp; platform-specific modes may vary	Substitution-dominant errors	Similar to other short-read platforms, limited ability to resolve plasmids, repeat-rich regions, and complex MGE structures without long-read support	Li et al., 2019; Kim et al., 2021
Oxford Nanopore Technologies (ONT)	Long to ultra-long reads, portability, rapid or real-time sequencing potential, and ability to improve resolution of plasmids, structural variants, and MGE-associated AMR context when read depth, chemistry, and analysis workflow are appropriate	Commonly ∼5 to > 100 kb, depending on DNA quality, library preparation, and run conditions	Insertions, deletions, and substitutions, with improved accuracy in recent chemistries and basecalling models	Per-base accuracy remains sensitive to chemistry, basecaller model, polishing strategy, and software version; conservative SNP-level interpretation may still require validation	Wick et al., 2017a; Jain et al., 2018; De Maio et al., 2019; Mantere et al., 2019

This table summarizes sequencing strategies according to their relevance to AMR interpretation rather than providing a platform-performance ranking. Selection should be guided by the surveillance question, required genomic resolution, validated local workflow, and the need for plasmid or mobile genetic element context. Detailed QC and preprocessing tools are summarized in Supplementary Table 1; short-read, long-read, and hybrid assembly workflows are summarized in Supplementary Tables 2–4; and additional alignment, basecalling, polishing, variant-calling, and mobile genetic element analysis tools are summarized in Supplementary Table 6. HiFi, high-fidelity circular consensus sequencing; ONT, Oxford Nanopore Technologies; MGE, mobile genetic element; ARG, antimicrobial-resistance gene.

In practice, WGS-based AMR analysis relies on short-read, long-read, or hybrid sequencing workflows, selected according to whether surveillance is focused primarily on SNP-level inference or on structural reconstruction of plasmids, transposons, and other MGEs (Bentley et al., 2008; Eid et al., 2009; Wick et al., 2017b; Wenger et al., 2019; Logsdon et al., 2020; Khezri et al., 2021; Benoit et al., 2024). Short-read sequencing-by-synthesis platforms, particularly Illumina platforms, offer scalable throughput and high base-call accuracy under appropriate quality-control conditions, rendering them suitable for the detection of resistance-associated variants and many acquired ARGs. However, limited read length can fragment assemblies and reduce resolution of repeat-rich regions, plasmid structures, and MGE architectures that are important for transmission analysis and mobilome interpretation (Eid et al., 2009; Jain et al., 2018; Wenger et al., 2019; Nurk et al., 2020; Benoit et al., 2024). The MGI DNBSEQ platforms have shown broadly comparable performance to Illumina in selected WGS and transcriptomic evaluations, although performance remains platform-, chemistry-, and workflow-dependent (Kim et al., 2021; Bestard-Cuche et al., 2024; Hu et al., 2024c). As a result, short-read platforms remain commonly used for large-scale SNP-based surveillance and routine presence/absence screening of many ARGs, although they remain less reliable for plasmid reconstruction, composite transposon resolution, and copy-number inference (Nagarajan and Pop, 2013; Khezri et al., 2021).

Long-read sequencing platforms, particularly PacBio HiFi and Oxford Nanopore Technologies (ONT), provide important advantages when AMR interpretation depends on structural genomic context (Eid et al., 2009; Jain et al., 2018; Wenger et al., 2019; Nurk et al., 2020). PacBio HiFi should be distinguished from older PacBio continuous long-read (CLR) workflows because HiFi reads are generated through circular consensus sequencing and generally support higher per-read accuracy with fewer downstream correction requirements. This makes PacBio HiFi particularly useful for near-complete bacterial genome reconstruction, plasmid resolution, and interpretation of repeat-rich resistance regions, provided that sequencing depth, DNA quality, library preparation, and assembly validation are adequate (Luan et al., 2024). ONT workflows have also improved with recent pore chemistries, library kits, and basecalling models, including R10.4.1/Kit14 and Dorado-based workflows. However, ONT performance remains chemistry-, model-, version-, and polishing-dependent; therefore, ONT-derived SNP calls, homopolymer-associated indels, and small variants in repetitive regions should be interpreted conservatively unless supported by adequate coverage, version-controlled workflows, and validation (Lemon et al., 2017; Bogaerts et al., 2024; Dabernig-Heinz et al., 2024; Hall et al., 2024; Sanderson et al., 2024). Thus, PacBio HiFi and ONT are best viewed as complementary long-read strategies with different error profiles and operational trade-offs rather than interchangeable technologies.

These structural advantages are especially important for understanding how AMR determinants are organized and disseminated. Long reads facilitate the association of ARGs with their respective carriers, which include plasmids, integrative conjugative elements (ICEs), and transposons. This advancement enables the detection of chromosomal integrations, copy-number variations, and rearrangements that are frequently challenging to resolve with short-read sequencing alone. These advantages are particularly important for complex resistance architectures, including *Klebsiella pneumoniae* carbapenemase (KPC)-linked transposons, such as Tn4401-related components, IS26-driven amplifications, and layered mobility patterns that influence the development and dissemination of multidrug resistance (Aihara et al., 2024; Mathers et al., 2024).

Hybrid sequencing strategies are especially valuable when both base-level precision and structural context are required (Koren et al., 2012; Wick et al., 2017a, b; De Maio et al., 2019; Khezri et al., 2021; Lipworth et al., 2024). By combining long-read continuity with short-read polishing accuracy, hybrid workflows can improve plasmid and transposon reconstruction, refine placement of co-located resistance determinants, and enhance discrimination of β-lactamase variants compared with either sequencing modality alone (Wick et al., 2017a; Khezri et al., 2021; Di Pilato et al., 2023; Lipworth et al., 2024). They may also clarify movement of ARGs between plasmids and chromosomes and identify configurations such as multicopy KPC-associated transposon arrangements, which are relevant to MGE epidemiology and comparable genomic interpretation for surveillance (Aihara et al., 2024; Zhang F. et al., 2025). These gains, however, depend on balanced sequencing depth, careful correction and polishing, and greater operational complexity and cost. Although the emergence of more accurate long-read chemistries, including PacBio HiFi and recent ONT R10/Kit14 workflows with contemporary basecalling, has reduced reliance on hybrid strategies in some settings (Espinosa et al., 2024), benchmarking studies indicate that, in selected bacterial workflows and at appropriate sequencing depth, hybrid assemblies can outperform single-platform approaches by combining long-read structural advantages with short-read base accuracy (De Maio et al., 2019; Espinosa et al., 2024). Accordingly, hybrid assembly remains valuable when both structural context and conservative base-level accuracy are required, but it should not be presented as a universal default. Workflow choice should be guided by the organism, sequencing platform, required resolution, validation framework, and intended AMR-reporting context.

## 3 AMR prediction tools and WGS-based interpretation workflows

Recent advances in bioinformatics have expanded the analytical value of WGS. However, in AMR surveillance, the key issue is not data processing alone, but whether outputs are reproducible, comparable, and interpretable for public health or clinical use. Because AMR inference depends on sequencing chemistry, software versions, parameter settings, input type, and database content, workflows should be version-pinned, benchmarked, and selected according to the surveillance objective.

This section focuses on workflow decision points that directly affect AMR interpretation, including platform-aware quality control, contamination screening, read-based versus assembly-based analysis, post-assembly assessment, and database-supported resistance reporting. Detailed QC and preprocessing tools are summarized in Supplementary Table 1; short-read, long-read, and hybrid assembly workflows are summarized in Supplementary Tables 2–4; and broader genome-analysis tools, including alignment, basecalling, polishing, variant calling, visualization, phylogenetic context, and mobile genetic element analysis, are summarized in Supplementary Table 6. AMR-specific prediction software and reference databases are summarized separately in Table 2 to distinguish resistance-gene detection and chromosomal mutation inference from general genome-analysis support tools. Figure 2 provides an AMR-focused overview of workflow decision points from sequencing data to resistance interpretation. Accordingly, the main text emphasizes only those workflow decisions that influence resistance detection, genotype–phenotype concordance, validation, and surveillance reporting.

**TABLE 2:** Comparative summary of AMR prediction tools and reference databases, including key strengths, intended applications, limitations, and potential sources of discordance.

Resource/tool	Primary function and key strength	Input type	Analytical strategy	Intended application(s)	Main interpretive limitation/potential source of discordance	References
ResFinder	Detection of acquired AMR genes	Assembled sequences and, depending on implementation, selected short-read-based workflows	Similarity-based sequence search against curated acquired ARG references	Isolate-based AMR screening in clinical, veterinary, and foodborne surveillance	Differences may arise from database version, identity/coverage thresholds, partial or mosaic alleles, and lack of mutation interpretation without PointFinder	Bortolaia et al., 2020; Florensa et al., 2022
CARD/RGI	Mechanism-aware AMR gene, allele, and mutation interpretation using the Antibiotic Resistance Ontology	Reads, contigs, predicted proteins or assemblies, depending on RGI workflow	Ontology-linked model-based detection, including curated resistance models and hit categories	Isolate and research workflows requiring mechanism-aware AMR interpretation	Interpretation can differ from threshold-based tools; low-confidence hits require cautious interpretation	Jia et al., 2017; Alcock et al., 2020, 2023
PlasmidFinder	Plasmid replicon detection and typing	Assembled contigs or compatible sequence input	BLAST-based replicon typing	Isolate-based plasmid epidemiology, especially in Enterobacterales	Replicon detection does not prove ARG localization on plasmids and does not resolve insertion sequences or transposons	Carattoli et al., 2014
AMRFinderPlus	Detection of acquired AMR genes, selected chromosomal resistance mutations, stress-response genes, and virulence-associated genes	Assembled nucleotide sequences, protein sequences, or annotated genomes	Curated protein/gene search with HMM-supported detection and selected organism-specific mutation models	Isolate-based clinical, public health, foodborne, and veterinary surveillance	Outputs depend on organism coverage, NCBI-curated reference sets, software/database version, and reporting thresholds	Feldgarden et al., 2019, 2021
PointFinder	Detection of curated chromosomal point mutations associated with AMR	Assembled contigs or compatible sequence data through CGE workflows	Organism-specific mutation detection in predefined resistance-associated loci	Isolate-based mutation inference for selected bacterial taxa	Limited to curated species–drug panels; lacking a panel does not rule out mutation-driven resistance.	Zankari et al., 2017
NDARO	Surveillance-linked AMR genotype, selected phenotype associations, and metadata integration	Public genome submissions and associated metadata	NCBI-linked AMR detection and surveillance data integration	Public health surveillance and comparative pathogen genomics	Coverage and phenotype-linked information vary by organism, sector, and submitted metadata quality	National Center for Biotechnology Information, 2023
SARG/ARGs-OAP	Environmental ARG annotation and abundance profiling	Metagenomic reads	Structured ARG database with HMM/sequence-based environmental resistome profiling	Environmental, wastewater, agricultural, and ecological AMR surveillance	Better suited to environmental/metagenomic analysis than direct clinical isolate interpretation	Yin et al., 2018
MEGARes/AMR++	Metagenomic AMR gene classification and resistome profiling	Metagenomic reads	Read alignment and ontology-based AMR classification	Environmental, agricultural, wastewater, and host-associated metagenomic resistome analysis	Not optimized for isolate-level clinical interpretation or chromosomal SNP-based resistance inference	Lakin et al., 2017; Doster et al., 2020; Bonin et al., 2023
ARGminer	Literature-linked AMR gene and mutation curation	Sequence queries or database lookup	Literature-supported curation, text mining, and sequence-based ARG identification	Supplementary AMR annotation in research and surveillance workflows	Curation depth and update consistency may vary; best used alongside established curated AMR resources	Arango-Argoty et al., 2020
BacMet	Detection and annotation of bacterial metal and biocide resistance genes	Sequence queries, genomes, or metagenomic data, depending on the workflow	Reference database search for metal and biocide resistance determinants	Environmental AMR, co-selection analysis, and One Health surveillance contexts	Complements antibiotic-resistance analysis but is not a core antibiotic ARG database	Pal et al., 2014
DeepARG	Machine-learning-assisted ARG prediction	Metagenomic reads or predicted gene sequences, depending on workflow	Deep-learning-based ARG classification supported by sequence-similarity features	Discovery-oriented and metagenomic AMR profiling	Model-dependent predictions require external validation and should not replace curated clinical interpretation	Arango-Argoty et al., 2018
BV-BRC (formerly PATRIC)	Broad bacterial and viral comparative genomics platform with AMR-associated analysis functions	Genome assemblies and associated metadata	Comparative genomics, annotation, AMR feature analysis, and platform-based workflows	Pathogen genomics, comparative analysis, and surveillance-oriented research	Broad genomics platform; not a specialized AMR reference database equivalent to CARD, ResFinder, or AMRFinderPlus	Wattam et al., 2017; Shukla et al., 2026
ARG-ANNOT	Curated ARG annotation, especially classical acquired resistance genes, including β-lactamases	Assembled contigs or sequence queries	Sequence-similarity search against curated ARG references	Isolate-based ARG annotation and supplementary confirmation	Legacy resource with more limited update continuity compared with newer curated databases	Gupta et al., 2014
SRST2	Read-based detection of resistance genes and alleles directly from short-read data	FASTQ short reads	Read mapping to reference gene or allele databases	Isolate-based AMR workflows where assembly is not required	Limited to known references; performance depends on read depth, allele similarity, and reference quality	Inouye et al., 2014
ARIBA	Read-based local assembly and variant-aware AMR detection	FASTQ short reads	Local assembly and mapping around target genes/loci	Isolate-based AMR detection with allele-level evidence from reads	Requires curated reference sets and sufficient read support; less suited for broad plasmid/MGE reconstruction	Hunt et al., 2017
ABRicate*	Screening of assembled contigs against selectable AMR databases	Assembled contigs	BLAST-based contig screening against databases such as ResFinder, CARD, ARG-ANNOT, NCBI, or MEGARes	Isolate-based assembled-genome screening	Dependent on assembly quality, selected database, and identity/coverage thresholds; does not detect mutational resistance from raw reads	Torsten Seemann and Björn Grüning, 2016
SSTAR	Stand-alone acquired AMR gene prediction	Assembled genome sequences or sequence files	Sequence-based screening against curated AMR gene references	Isolate-based ARG screening and supplementary AMR annotation	Best interpreted as a screening tool; results depend on reference database and cutoff settings	de Man and Limbago, 2016

*ABRicate is a software utility rather than an AMR database; therefore, its official repository and the specific database used should be cited. CARD, comprehensive antibiotic-resistance database; BLAST, basic local alignment search tool; BV-BRC, Bacterial and Viral Bioinformatics Resource Center; PATRIC, Pathosystems Resource Integration Center; NDARO, National Database of Antibiotic-Resistant Organisms; SARG, Structured antibiotic resistance genes ARGs; MEGARes, Metagenomic Antimicrobial Resistance database; ARG-ANNOT, Antibiotic Resistance Gene-ANNOTation.

### 3.1 Quality-control requirements for reliable WGS-AMR inference

Quality control is essential for WGS-AMR interpretation because poor read quality, contamination, uneven coverage, or platform-specific errors can alter gene detection, mutation calling, and downstream genotype–phenotype interpretation (Cock et al., 2009; Ewels et al., 2016; Low et al., 2019; Wood et al., 2019). However, QC is treated here as an AMR-relevant decision checkpoint rather than as an independent focus of the review. In practical AMR workflows, FASTQ-format quality scores provide the basis for read-level quality assessment (Cock et al., 2009), while MultiQC summarizes outputs across multiple tools and samples (Ewels et al., 2016).

Quality control criteria should be selected according to the intended AMR workflow. Read-based tools such as SRST2 and ARIBA require sufficient read depth, coverage breadth, and allele-level support (Inouye et al., 2014; Hunt et al., 2017), whereas contig-based screening tools such as ABRicate depend more strongly on assembly quality, contig fragmentation, selected database, and identity/coverage thresholds (Torsten Seemann and Björn Grüning, 2016). Long-read or hybrid workflows require additional attention to sequencing chemistry, basecaller model, polishing strategy, and structural validation (Lemon et al., 2017; Bogaerts et al., 2024; Dabernig-Heinz et al., 2024; Luan et al., 2024; Sanderson et al., 2024), especially when AMR interpretation depends on plasmid reconstruction, transposon structure, or linkage between resistance genes and other MGEs (Carattoli et al., 2014; Arredondo-Alonso et al., 2017; Wick et al., 2021, 2023). Supplementary Table 1 summarizes detailed QC, trimming, contamination-screening, and platform-specific preprocessing tools, while Supplementary Table 6 summarizes broader genome-analysis support tools.

### 3.2 Read-mapping, assembly, and structural context in AMR interpretation

Whole-genome sequencing-based AMR workflows should be selected according to the biological question, rather than assuming that *de novo* assembly is required for all resistance analyses. Many isolate-based AMR tools operate directly on FASTQ reads through read mapping or local assembly, including SRST2 and ARIBA, and can detect known acquired resistance genes, alleles, and selected resistance-associated variants while retaining interpretable support metrics such as read depth, coverage breadth, alignment quality, and allele balance (Inouye et al., 2014; Hunt et al., 2017). These read-based approaches are particularly useful for routine surveillance when the primary objective is the detection of known ARGs or curated variants rather than the reconstruction of complete genomes. Contig-based tools such as ABRicate remain useful when assembled genomes are already available, but their outputs depend on assembly quality, selected database, and identity/coverage thresholds (Torsten Seemann and Björn Grüning, 2016). Therefore, assembly-based AMR analysis should not be treated as the default gold standard; it is most informative when the biological question requires genomic context, such as plasmid reconstruction, MGE localization, copy-number structure, or linkage between resistance genes and mobile elements (Carattoli et al., 2014; Arredondo-Alonso et al., 2017; Wick et al., 2021, 2023).

When assembly is required, bacterial genomes can be reconstructed either through reference-guided approaches or through *de novo* assembly, depending on the study objective, sequencing platform, and required level of structural resolution (Palmer et al., 2010; Ji et al., 2011; Lischer and Shimizu, 2017). These factors determine achievable contiguity, structural resolution, and downstream interpretability. Representative short-read, long-read, and hybrid assembly workflows are summarized in Supplementary Tables 2–4, respectively.

#### 3.2.1 Short-read assembly

Short-read assembly should be discussed as one support layer for AMR interpretation, not as a required step for all AMR detection workflows. When the objective is routine detection of known acquired ARGs or alleles, read-based tools such as SRST2 and ARIBA may provide direct FASTQ-level evidence without whole-genome assembly (Inouye et al., 2014; Hunt et al., 2017).

Short-read assembly remains useful in bacterial WGS, although its main limitation is the difficulty of resolving repetitive genomic regions. de bruijn graph (DBG) assemblers such as SPAdes reconstruct genomes efficiently from datasets such as 2 × 150 bp reads by using multi-k iterations and error-correction steps to improve contiguity and reduce misassembly (Bankevich et al., 2012). Although Illumina platforms provide several read-length configurations and chemistries, improvements in repeat resolution remain modest. Library preparation is also important, since PCR-based workflows can introduce GC-related bias, whereas PCR-free protocols improve coverage uniformity (Illumina, 2024). For large or cohort-scale datasets, assemblers such as MEGAHIT use compact graph representations and memory-efficient heuristics to reduce runtime and RAM demands while maintaining acceptable assembly quality (Li et al., 2015).

For surveillance applications, short-read WGS remains well-suited to variant-centric analyses, including SNP-based phylogenetic inference and detection of many acquired resistance genes and point mutations, particularly when standardized mapping and variant-calling workflows are combined with curated reference resources. This makes short reads well-suited for large-scale monitoring of clonal spread and for routine screening workflows in which the primary objective is lineage assignment and determinant detection rather than complete reconstruction of plasmids or other MGEs (Ellington et al., 2017; Armstrong et al., 2019; Mahfouz et al., 2020; Ferdinand et al., 2021; Sherry et al., 2025).

The main trade-offs in short-read assembly involve *k*-mer choice, insert-size calibration, coverage distribution, and repeat structure. Larger *k*-mers improve specificity but may fragment low-coverage regions, whereas smaller *k*-mers can bridge coverage gaps at the expense of increasing chimeric assemblies around insertion sequences, prophages, multicopy loci, and plasmids. As a result, plasmid-rich and repeat-rich loci are frequently unresolved when short reads are used alone, limiting reconstruction of AMR genetic context unless long-read or hybrid approaches are incorporated (Arredondo-Alonso et al., 2017). This becomes especially important when interpretation depends on linkage, for example, whether multiple resistance genes are co-located on the same plasmid, whether a β-lactamase is embedded within a particular transposon, or whether IS-mediated amplification is present. In such settings, assembly fragmentation can disrupt these structures across contigs and obscure junction evidence and copy-number signals. Consequently, short-read assemblies may support reliable gene presence or absence calls while still providing incomplete information about mobility, co-selection potential, and copy-number-dependent effects relevant to transmission and phenotype interpretation (Ellington et al., 2017; Armstrong et al., 2019; Ferdinand et al., 2021; Sherry et al., 2025).

Assembly assessment should therefore not rely solely on continuity metrics. Objective evaluation should combine structural assessment, such as QUAST/WebQUAST-derived measures including NGA50 and misassembly counts, with mapped-read support and microbial genome-quality assessment where relevant (Gurevich et al., 2013; Parks et al., 2015; Chklovski et al., 2023; Mikheenko et al., 2023). BUSCO may provide optional marker-gene completeness information, but it should not be treated as a substitute for microbial contamination assessment (Waterhouse et al., 2018; Seppey et al., 2019). SPAdes remains commonly used for bacterial short-read assembly because of its multi-k strategy and integrated error-correction approach, although computational requirements and output quality remain dataset-dependent (Bankevich et al., 2012). In practice, closed bacterial genomes are rarely achieved using short reads alone. Genome closure typically requires long reads with sufficient consensus accuracy, such as PacBio HiFi or polished ONT data or, alternatively, a hybrid workflow (Wenger et al., 2019; Nurk et al., 2020; Sanderson et al., 2024). Depth alone is not sufficient: many isolates can be closed with approximately 20–40 × long-read coverage, often with short-read polishing, whereas even ∼100 × short-read coverage rarely resolves complex repeats or guarantees closure (De Maio et al., 2019; Khezri et al., 2021). For plasmid-context interpretation, long-read sequencing combined with dedicated tools such as MOB-suite and PlasCAT, and with PlasmidFinder for replicon typing, can provide stronger evidence for plasmid and MGE context than short-read-only workflows, particularly in complex plasmidomes (Carattoli et al., 2014; Robertson and Nash, 2018; Peccoud et al., 2024).

Overall, short-read assembly remains a commonly used and scalable option for routine surveillance and resistome screening, but its interpretive limits should be recognized. Although legacy short-read assemblers such as Velvet were important in the development of de Bruijn graph assembly, they should not be presented as current default recommendations for bacterial WGS-AMR workflows (Zerbino and Birney, 2008; Zerbino, 2010). Contemporary short-read workflows more commonly use actively maintained assemblers such as SPAdes or SKESA, while long-read or hybrid strategies are preferred when AMR interpretation depends on plasmid reconstruction, repeat resolution, or MGE context (Zerbino and Birney, 2008; Bankevich et al., 2012; Carattoli et al., 2014; Arredondo-Alonso et al., 2017; Souvorov et al., 2018). Accordingly, when surveillance conclusions depend on structural genomic context and conservative base-level accuracy, hybrid strategies remain valuable, although workflow choice should be guided by organism, platform, depth, validation requirements, and reporting context.

#### 3.2.2 Long-read assembly

Long-read sequencing can improve assembly contiguity and structural interpretation by spanning repetitive regions, plasmids, and other complex loci that are often unresolved with short reads alone (Arredondo-Alonso et al., 2017; Wick et al., 2023; Sanderson et al., 2024). In bacterial AMR genomics, its main value is not simply producing longer contigs, but improving the ability to place resistance determinants within plasmids, transposons, repeat-rich regions, and chromosomal contexts (Carattoli et al., 2014; Lemon et al., 2017; Aihara et al., 2024; Lipworth et al., 2024). PacBio HiFi and ONT should be discussed separately because they differ in read-generation chemistry, error profile, polishing requirements, and operational assumptions (Wenger et al., 2019; Luan et al., 2024; Sanderson et al., 2024). PacBio HiFi provides high-consensus accuracy through circular consensus sequencing, whereas ONT offers flexible read lengths and rapid or real-time sequencing potential, but remains more sensitive to chemistry, basecaller model, polishing strategy, and software version (Wenger et al., 2019; Bogaerts et al., 2024; Dabernig-Heinz et al., 2024; Sanderson et al., 2024). These differences should be reported explicitly when long-read data are used for AMR interpretation (Dabernig-Heinz et al., 2024; Luan et al., 2024; Sanderson et al., 2024).

*De novo* long-read assembly typically includes read processing, assembly, and post-assembly polishing, with workflow choice influenced by the sequencing platform and expected error profile (Koren et al., 2012; Hu et al., 2024b; Zhang E. et al., 2025). Representative long-read assemblers include Canu/HiCanu, Raven, Flye, and wtdbg2, with additional tool-level comparisons provided in Supplementary Table 3 (Kolmogorov et al., 2019; Nurk et al., 2020; Ruan and Li, 2020; Shafin et al., 2020; Cheng et al., 2021; Vaser and Šikiæ, 2021). Polishing and validation remain important because platform-specific errors, particularly in homopolymers and low-complexity regions, can affect downstream AMR interpretation (Walker et al., 2014; Vaser et al., 2017; Hu et al., 2020; Shafin et al., 2020; Huang et al., 2021). Final assembly quality, therefore, depends not only on read type and assembler choice, but also on the polishing strategy and the rigor of subsequent QC.

Long-read assembly is commonly implemented through overlap-based or graph-based frameworks that generate consensus contigs from noisy reads (Sohn and Nam, 2018). Error correction may rely on self-consensus or hybrid support, although hybrid correction can introduce bias in GC-rich or unevenly covered regions and may suppress genuine variants if applied uncritically (Sohn and Nam, 2018). For this reason, polishing parameters and workflow configuration should be selected carefully, because inappropriate choices can reinforce systematic rather than random errors. These considerations are especially important when assemblies will be used for SNP-based inference or detailed interpretation of AMR-associated loci.

Recent advancements in ONT chemistry, library preparation, basecalling, and polishing have enhanced the applicability of nanopore sequencing in bacterial genomics; however, performance remains workflow-specific. Dorado is commonly used in recent ONT workflows and supports simplex, duplex, and modified-base workflows; however, performance depends on pore chemistry, library kit, basecalling model, software version, read depth, and downstream polishing strategy. When combined with R10.4.1 pores, Kit 14, and contemporary super-accuracy (SUP) basecalling or duplex workflows, Dorado-based analyses can improve read accuracy and reduce indel burden compared with earlier ONT generations, although performance remains dependent on chemistry, model, software version, coverage, and polishing strategy (Bogaerts et al., 2024; Dabernig-Heinz et al., 2024; Sanderson et al., 2024). Nevertheless, ONT-only workflows should not be treated as universally equivalent to hybrid assembly. Their suitability depends on coverage depth and uniformity, organism and genome structure, polishing choices, and the intended reporting context (Wick et al., 2023; Luan et al., 2024; Sanderson et al., 2024). Homopolymer-associated indels, small-variant discordance in repetitive motifs, and version-to-version changes in basecalling behavior remain relevant concerns (Bogaerts et al., 2024; Luan et al., 2024; Sanderson et al., 2024). For this reason, ONT-only AMR interpretation should be reported with full chemistry, kit, basecaller model, software version, coverage, polishing strategy, and validation details, particularly when SNP-level precision, longitudinal comparability, or clinical interpretation is required (Bogaerts et al., 2024; Dabernig-Heinz et al., 2024; Luan et al., 2024; Sanderson et al., 2024). Hybrid approaches remain valuable when reporting depends on conservative small-variant accuracy, reliable homopolymer resolution, strict longitudinal comparability, or high-confidence reconstruction of plasmid and MGE junctions (De Maio et al., 2019; Dabernig-Heinz et al., 2024; Luan et al., 2024). Thus, hybrid assembly should be a targeted, question-driven strategy rather than a universal default, with workflow selection guided by the organism, sequencing chemistry, coverage, validation requirements, and intended AMR-reporting context.

Consensus-of-assemblies approaches can further improve long-read assembly accuracy when high-confidence reconstruction is required. Tools such as Trycycler combine multiple assemblies from the same isolate, cluster homologous contigs, resolve discrepancies, and refine a consensus, often producing fewer SNPs, indels, and structural errors than a single-assembler workflow after polishing (Wick et al., 2021). Autocycler was developed to automate much of this process and generate a streamlined consensus assembly with less manual intervention (Wick et al., 2025). These approaches are most useful when accurate circularization of plasmids and AMR-associated MGEs is essential, although they still depend on sufficient long-read depth, effective polishing, and read-backed validation of key junctions (Wick et al., 2021, 2023).

#### 3.2.3 Hybrid assembly approaches

Hybrid assembly is valuable in AMR genomics when resistance interpretation requires both structural context and conservative base-level accuracy. By combining long-read continuity with short-read accuracy, hybrid workflows can improve the resolution of repetitive regions, support plasmid reconstruction and circularization, and improve placement of AMR determinants within plasmids, transposons, insertion sequences, or other MGEs (Koren et al., 2012; Wick et al., 2017b; Robertson and Nash, 2018). This is most relevant when the surveillance question depends on whether resistance genes are co-located on the same plasmid, embedded within a transposon, associated with repeat-mediated amplification, or linked to other mobile elements.

Hybrid workflows, such as Unicycler, combine long-read contiguity with short-read accuracy to improve recovery of plasmid and MGE structures relevant to downstream plasmid typing and plasmidome analysis (Koren et al., 2012; Carattoli et al., 2014; Wick et al., 2017b; Robertson and Nash, 2018). However, hybrid assembly should not be presented as a universal requirement for AMR analysis. It is most useful when read-based or contig-based AMR detection alone cannot answer the biological question, particularly when structural linkage, plasmid localization, or MGE context is required. Representative hybrid assembly, scaffolding, and correction workflows, together with their main interpretation limits, are summarized in Supplementary Table 4.

Hybrid correction and post-assembly polishing should be distinguished. Hybrid correction uses accurate short reads to correct long reads before assembly, whereas polishing is applied after contigs have already been generated. Methods such as LoRDEC, proovread, Ratatosk, FMLRC2, and HERO can improve per-base accuracy or reduce indel burden in selected workflows, but performance depends on sequencing depth, genome complexity, coverage balance, and computational constraints (Hackl et al., 2014; Salmela and Rivals, 2014; Zhang et al., 2020; Holley et al., 2021; Kang et al., 2023; Mak et al., 2023; Muñoz-Barrera et al., 2025). Because overly aggressive correction may suppress true biological variation in biased or low-coverage regions, correction and polishing parameters should remain task-specific and supported by validation when AMR reporting depends on SNP-level precision, plasmid junctions, or structural correctness (Zhang et al., 2020; Muñoz-Barrera et al., 2025).

#### 3.2.4 Post-assembly assessment: contiguity and structural integrity

After assembly, reproducible post-assembly evaluation is essential before contigs are used as reliable evidence for downstream AMR interpretation. Assembly quality should not be judged by a single continuity metric such as N50. For bacterial assemblies, microbial genome-quality frameworks such as CheckM or CheckM2 are more appropriate for assessing completeness and contamination (Parks et al., 2015; Chklovski et al., 2023). QUAST/WebQUAST, read-mapping evidence, circularization status, and plasmid validation provide complementary information on contiguity and structural correctness, including misassembly counts, NA50/NGA50, and indel profiles (Gurevich et al., 2013; Mikheenko et al., 2023). BUSCO may be retained as an optional secondary marker-gene completeness indicator, but it should not be treated as a substitute for microbial contamination assessment (Waterhouse et al., 2018; Seppey et al., 2019). Where available, reference-free consensus accuracy estimates such as Merqury QV can further strengthen assembly evaluation, although such approaches depend on the availability of appropriate *k*-mer evidence and should be interpreted alongside mapped-read support and targeted structural checks (Rhie et al., 2020). Accordingly, assembly assessment should combine continuity, completeness, contamination, and structural-validation evidence, particularly when downstream AMR interpretation depends on plasmid, MGE, or resistance-locus context.

For AMR interpretation, assembly assessment should focus on whether the resistance-relevant structures are preserved. Fragmentation, residual indels, misassemblies, or loss of short low-coverage contigs may separate ARGs from plasmids, transposons, insertion sequences, or other MGEs, thereby limiting interpretation of gene mobility, copy number, and genotype–phenotype concordance (Lantz et al., 2018; Bertrand et al., 2019; Hu et al., 2024c). Therefore, continuity metrics should be reported together with contamination estimates, structural correctness, circularization evidence, plasmid/MGE validation, mapped-read support, and AMR-specific downstream requirements rather than interpreted in isolation (Gurevich et al., 2013; Parks et al., 2015; Mikheenko et al., 2018, 2023; Waterhouse et al., 2018; Seppey et al., 2019; Rhie et al., 2020; Chklovski et al., 2023).

Polishing and validation should be interpreted according to sequencing chemistry and reporting context. ONT assemblies may require ONT-specific polishing to reduce indel and homopolymer-associated errors, whereas PacBio HiFi assemblies often require fewer corrections but should still be checked at AMR-relevant loci, plasmid junctions, and repetitive regions (Walker et al., 2014; Warren et al., 2019; Hu et al., 2020, 2024a; Zimin and Salzberg, 2020; Huang et al., 2021; Wick and Holt, 2022; Hall et al., 2024). Short-read polishing may improve residual single-nucleotide and micro-indel errors in long-read assemblies, but polishing order and tool choice can affect final accuracy. Therefore, results should be validated when AMR reporting depends on SNP-level precision or structural correctness (Walker et al., 2014; Warren et al., 2019; Hu et al., 2020, 2024a; Zimin and Salzberg, 2020; Huang et al., 2021; Wick and Holt, 2022). Additional details on QC, preprocessing, assembly-level quality assessment, polishing, alignment, basecalling, and related genome-analysis tools are provided in Supplementary Tables 1, 6, with assembly-specific workflows summarized in Supplementary Tables 2–4. These workflow distinctions provide the analytical context for selecting AMR prediction tools and reference databases according to input type, organism, resistance mechanism, and reporting purpose, as discussed in section 3.3.

### 3.3 Antimicrobial resistance prediction software and reference databases

Building on the workflow distinctions described in section 3.2, selection of AMR software and reference databases should be guided by input type, organism, resistance mechanism, reporting purpose, and the need for genomic-context interpretation. Read-based and local-assembly approaches can provide direct evidence for known resistance genes, alleles, and selected mutations without whole-genome assembly (Inouye et al., 2014; Hunt et al., 2017), whereas contig-based approaches are useful when assembled genomes and gene-context analysis are required (Torsten Seemann and Björn Grüning, 2016; Arredondo-Alonso et al., 2017). For plasmid- or MGE-associated resistance, AMR gene calls should be interpreted with plasmid typing and, where necessary, long-read or hybrid structural evidence (Carattoli et al., 2014; Arredondo-Alonso et al., 2017; Robertson and Nash, 2018; Khezri et al., 2021). Metagenomic resistome workflows should be considered separately because mixed-community data require different abundance models, analytical thresholds, contamination controls, and validation frameworks from those used for isolate-based WGS (Yin et al., 2018; Lee et al., 2021; Bonin et al., 2023). The corresponding workflow trade-offs are detailed in section 3.2 and summarized in Table 2.

AMR surveillance increasingly relies on specialized reference databases designed for different analytical purposes rather than on a single generic resource. Whether a single consolidated ARG database would improve AMR interpretation remains uncertain; a federated model may better preserve context-specific curation while improving interoperability. Instead, global AMR surveillance would benefit more from federated interoperability based on harmonized ontologies, explicit evidence grading, version-controlled crosswalks, and independent benchmarking. Such an approach would preserve the strengths of context-specific resources, including clinically curated catalogues, mutation-focused repositories, and broader mechanism-oriented databases, while improving transparent and comparable reporting across One Health domains. At present, however, the database landscape remains fragmented. Resources differ in ontology, curation policy, evidence grading, update frequency, licensing, and treatment of alleles, mutations, and genomic context. These differences can lead to discordant calls, inconsistent interpretation of gene and allele assignments, variable treatment of MGEs, and uneven genotype–phenotype mapping, thereby limiting cross-study comparability and, in some settings, stewardship relevance (Lee et al., 2021; Papp and Solymosi, 2022). For this reason, database evaluation should extend beyond size alone and consider curation depth, versioning and sustainability, algorithmic compatibility, vocabulary interoperability, and metadata quality, as well as common pitfalls such as permissive homology thresholds, redundant entries, and incomplete provenance.

Among commonly used isolate-focused AMR tools, AMRFinderPlus, ResFinder/PointFinder, CARD/RGI, SRST2, ARIBA, and ABRicate serve complementary rather than interchangeable functions. AMRFinderPlus supports NCBI-curated detection of acquired resistance genes and selected chromosomal resistance mutations (Feldgarden et al., 2019, 2021). ResFinder identifies acquired AMR genes, while PointFinder provides curated mutation-based resistance inference for selected bacterial taxa (Zankari et al., 2017; Bortolaia et al., 2020; Florensa et al., 2022). CARD/RGI applies ontology-linked resistance models for mechanism-aware interpretation (Jia et al., 2017; Alcock et al., 2020, 2023). SRST2 and ARIBA provide read-based or local-assembly genotyping directly from short-read data (Inouye et al., 2014; Hunt et al., 2017; Cooper et al., 2020), whereas ABRicate screens assembled contigs against user-selected databases and is therefore sensitive to database selection, identity and coverage thresholds, and assembly quality (Torsten Seemann and Björn Grüning, 2016). Accordingly, tool selection should be matched to the organism, input data type, resistance mechanism, required genomic context, and intended clinical or surveillance use, with software versions, database versions, and decision thresholds reported transparently.

A practical AMR detection workflow should therefore begin with the biological and surveillance question rather than with a fixed assembly strategy. For routine isolate screening, read-based or contig-based tools may be sufficient for detecting common acquired ARGs and curated mutations. For mutation-mediated resistance, organism-specific mutation resources and careful genotype–phenotype benchmarking are required. For plasmid-mediated or MGE-associated resistance, AMR gene calls should be linked to plasmid replicon typing, long-read or hybrid assembly evidence, and junction-level validation where possible. For metagenomic or environmental surveillance, resistome tools need to operate under different assumptions, thresholds, and reporting frameworks compared to those used in single-colony isolate workflows. To support practical tool selection, Table 2 compares representative AMR resources according to their primary function and key strength, input requirements, analytical strategy, intended application, and principal sources of interpretive discordance. Table 3 then synthesizes the broader analytical trade-offs and harmonization implications across these resource categories. Additional sector-specific surveillance applications of selected AMR resources across human, animal, and environmental settings are summarized in Supplementary Table 7.

**TABLE 3:** Analytical trade-offs and harmonization gaps across representative resources used in WGS-based AMR interpretation.

Analytical function/interpretation needs	Representative resources	Representative taxa or settings where relevant	Main trade-off or limitation	Harmonization implication	Representative supporting references
Acquired AMR gene detection	ResFinder, AMRFinderPlus, CARD	*Escherichia coli*, *Klebsiella pneumoniae*, *Salmonella enterica*, *Staphylococcus aureus*	Acquired gene detection is well-supported, but outputs may differ because of database scope, ontology, sequence thresholds, and treatment of partial or mosaic alleles	Cross-resource comparison requires explicit database versioning, threshold reporting, and standardized gene nomenclature	Alcock et al., 2020; Bortolaia et al., 2020; Feldgarden et al., 2021
Chromosomal mutation-based resistance inference	PointFinder, AMRFinderPlus, CARD	*Neisseria gonorrhoeae*, *Campylobacter jejuni*, *Helicobacter pylori*, *Mycobacterium tuberculosis*	Mutation support is taxon-dependent and often limited to curated organism-specific panels; absence of a curated panel should not be interpreted as absence of mutation-mediated resistance	Harmonization requires organism-specific mutation catalogues, shared evidence rules, and careful benchmarking against phenotypic AST	Zankari et al., 2017; CRyPTIC Consortium and the 100, 000 Genomes Project, 2018; Feldgarden et al., 2019; Gygli et al., 2019
Plasmid replicon typing and plasmid-associated dissemination	PlasmidFinder, MOB-suite, long-read or hybrid assembly workflows	*E. coli*, *K. pneumoniae*, *Enterobacter cloacae* complex, *S. enterica*	Replicon typing can support plasmid-backbone inference, but it does not, by itself resolve transposons, insertion sequences, ARG localization, or complete MGE architecture	Harmonized AMR interpretation requires plasmid context to be linked with ARG calls, structural evidence, and assembly quality	Carattoli et al., 2014; Arredondo-Alonso et al., 2017; Robertson and Nash, 2018; Khezri et al., 2021
MGE structural context and resistance gene neighborhood analysis	Long-read assembly, hybrid assembly, CARD, targeted plasmid/MGE tools	Plasmid-rich multidrug-resistant Enterobacterales, carbapenem-resistant *K. pneumoniae*, complex *Acinetobacter baumannii* isolates	Short-read workflows often detect genes without fully resolving structural context, copy number, or chromosomal versus plasmid location	Harmonization depends on distinguishing gene presence from genomic context and on transparent reporting of structural confidence	Carattoli et al., 2014; Arredondo-Alonso et al., 2017; Robertson and Nash, 2018; Khezri et al., 2021
Mechanism-aware interpretation and ontology-linked AMR annotation	CARD, AMRFinderPlus	Broad clinical pathogen workflows; mechanism-oriented interpretation across research and surveillance settings	Ontology-rich resources improve mechanistic interpretation, but may not always align with threshold-based gene calling tools or organism-specific mutation resources	Shared ontologies and crosswalks are needed to improve comparability between mechanism-based and threshold-based outputs	Alcock et al., 2020, 2023; Feldgarden et al., 2021
Routine surveillance-oriented isolate analysis	AMRFinderPlus, ResFinder, NDARO-linked workflows	Common hospital and public health pathogens, including *E. coli*, *K. pneumoniae*, *S. aureus*, *A. baumannii*	Routine workflows prioritize reproducibility and throughput, but may provide limited structural detail or organism-specific nuance if used without complementary resources	Harmonization requires version-controlled workflows, standardized reporting fields, and explicit handling of uncertainty	Bortolaia et al., 2020; Feldgarden et al., 2021; National Center for Biotechnology Information, 2023
Environmental and metagenomic resistome analysis	SARG, MEGARes, DeepARG, BacMet	Wastewater, soil, agriculture, aquaculture, mixed environmental samples	Environmental resources expand One Health surveillance but are not directly interchangeable with isolate-focused clinical databases; model-based tools may increase the number of uncertain or false-positive assignments if not carefully validated	Harmonization requires clear separation of isolate-level and metagenomic inference, together with standardized environmental metadata, and annotation rules	Pal et al., 2014; Arango-Argoty et al., 2018, 2020; Yin et al., 2018; Bonin et al., 2023
Co-selection and non-antibiotic resistance context	BacMet, environmental AMR workflows	Wastewater, agriculture, metal- or biocide-exposed environments	Non-antibiotic resistance determinants are important for co-selection but do not substitute for core AMR gene or mutation resources	Harmonized surveillance should incorporate co-selection context without conflating it with direct antibiotic resistance prediction	Pal et al., 2014
Integrated pathogen genomics and broader comparative analysis	BV-BRC, NDARO, broader NCBI-linked resources	Comparative pathogen genomics, surveillance integration, and broader research workflows	Broad platforms provide contextual genomic analysis, but they are not specialized substitutes for curated AMR reference databases	Harmonization benefits from linking dedicated AMR resources with broader pathogen genomics ecosystems rather than replacing one with the other	National Center for Biotechnology Information, 2023; Olson et al., 2023; Shukla et al., 2026
Clinically interpretable or high-stakes reporting	Combined use of curated AMR databases, mutation resources, plasmid/MGE context tools, and phenotypic AST	Bloodstream infection isolates, MDR hospital pathogens, tuberculosis, gonococcal AMR, outbreak investigations	No single resource is sufficient for clinically interpretable or surveillance-grade reporting across all taxa and mechanisms	Harmonization should prioritize multi-resource workflows, phenotype anchoring, auditable provenance, and organism-specific validation rather than reliance on a single universal database	Clausen et al., 2016; Ellington et al., 2017; Feldgarden et al., 2019; Rebelo et al., 2022
Detection, interpretation, validation, and reporting pathway	AMRFinderPlus, ResFinder/PointFinder, CARD/RGI, PlasmidFinder, AST/MIC systems, EUCAST/CLSI frameworks	All isolate-based WGS-AMR workflows and high-stakes surveillance contexts	Detection of a gene, allele, mutation, or plasmid marker does not itself establish phenotypic resistance or clinical actionability	Harmonized workflows should explicitly separate detected genomic features, interpreted resistance relevance, phenotype-linked validation, uncertainty category, and final reporting level	Clausen et al., 2016; Jia et al., 2017; Zankari et al., 2017; Burnham et al., 2017; Ellington et al., 2017; Feldgarden et al., 2019, 2021; Alcock et al., 2020, 2023; Bortolaia et al., 2020; Cooper et al., 2020; Rebelo et al., 2022; Simner et al., 2022; Florensa et al., 2022

This table provides a critical synthesis of analytical roles, practical trade-offs, and implications for harmonization; it is not intended as a ranking or measure of equivalent performance. Because the listed resources differ substantially in purpose, input requirements, and interpretation logic, they should be viewed as complementary rather than interchangeable components of WGS-based AMR workflows.

ResFinder remains a commonly used curated resource for identifying acquired resistance genes from WGS data, particularly from assembled sequences and selected short-read-based implementations. When paired with PointFinder, it also supports mutation-based resistance inference through curated gene catalogues and phenotype-mapping tables (Zankari et al., 2017; Bortolaia et al., 2020; Mahfouz et al., 2020). Together, these resources are useful for structured screening in research, surveillance, and diagnostic workflows. However, their outputs still depend on database content, update frequency, implementation, and user-defined thresholds. BLAST-based identification may misclassify truncated or mosaic alleles and may fail to detect novel variants, while contamination and assembly errors can generate misleading calls. ResFinder and PointFinder should therefore be used within validated, version-specific pipelines rather than as standalone proof of resistance, and clinically or epidemiologically important predictions should be confirmed using phenotypic AST or orthogonal evidence where appropriate (Clausen et al., 2016; Ellington et al., 2017; Papp and Solymosi, 2022; Rebelo et al., 2022).

AMRFinderPlus offers a different balance of strengths. Embedded within NCBI’s broader Pathogen Detection ecosystem, including the Reference Gene Catalog, Reference hidden Markov model (HMM) Catalog, and MicroBIGG-E outputs, it supports surveillance-oriented and versioned genomic interpretation. The tool uses a curated database of AMR proteins, hidden Markov models, and selected chromosomal resistance determinants, and it has shown strong genotype–phenotype concordance in evaluated isolate collections, although performance remains organism-, phenotype-, and database-version dependent (Feldgarden et al., 2021). Comparative evaluations indicate that AMRFinderPlus can provide complementary calls to other AMR resources, depending on organism, database version, and detection thresholds (Feldgarden et al., 2019, 2021). Nevertheless, discordance between AMRFinderPlus and other resources can still arise because of differences in ontology, inclusion criteria, identity and coverage thresholds, and treatment of mosaic or partial alleles. As with other tools, database version, software version, and relevant cutoffs should therefore be reported explicitly, and predictions with major clinical or surveillance implications should not be interpreted without context.

Complementary resources extend interpretation beyond core gene calling. PlasmidFinder supports plasmid replicon typing and can contribute to the interpretation of plasmid-associated dissemination, but it does not reconstruct plasmids or prove ARG localization (Carattoli et al., 2014). Other resources, including ARGminer, DeepARG, and MEGARes, support broader or more discovery-oriented detection of resistance determinants (Arango-Argoty et al., 2018, 2020; Bonin et al., 2023). MEGARes also provides derivative databases, cloud-compatible analytical workflows, and statistical frameworks that are particularly relevant for large-scale or environmental resistome studies (Gibson et al., 2015; Rowe et al., 2015; Lakin et al., 2017). However, broader discovery-oriented approaches may introduce additional risks of model bias, overcalling, or false positives, particularly when predictions are not supported by curated evidence or genomic context. Their outputs are therefore most reliable when incorporated into benchmarked, version-pinned pipelines and interpreted with orthogonal validation.

To move beyond database cataloguing and clarify why no single resource is sufficient for WGS-based AMR interpretation, Table 3 summarizes the principal analytical functions, trade-offs, and harmonization gaps across representative AMR resources and use contexts. Rather than treating database coverage as a simple organism-level matrix, this synthesis emphasizes that different resources serve distinct analytical purposes, including acquired gene detection, curated chromosomal mutation support, plasmid replicon typing, MGE contextualization, and environmental or metagenomic resistome analysis. Clinical pathogens such as *Escherichia coli*, *K. pneumoniae*, and *Staphylococcus aureus* are broadly represented in major resources, including CARD, AMRFinderPlus, and ResFinder (Feldgarden et al., 2019, 2021; Alcock et al., 2020; Bortolaia et al., 2020), whereas mutation-driven resistance in organisms such as *Mycobacterium tuberculosis* depends more heavily on curated variant catalogues and organism-specific mutation frameworks (CRyPTIC Consortium and the 100, 000 Genomes Project, 2018; Gygli et al., 2019). Similarly, plasmid-associated dissemination may be informed by tools such as PlasmidFinder, but plasmid replicon typing alone does not distinguish insertion sequences, transposons, or other transposable elements; these require more dedicated mobility-focused annotation and, ideally, junction-level validation (Carattoli et al., 2014). Environmental and metagenomic resources such as SARG, MEGARes, DeepARG, and BacMet extend AMR analysis beyond traditional clinical targets, but their outputs are not directly interchangeable with isolate-focused clinical databases (Gibson et al., 2015; Rowe et al., 2015; Lakin et al., 2017; Arango-Argoty et al., 2020). Additional organism-linked applicability details are summarized in Supplementary Table 5 as an illustrative graded summary rather than a binary coverage matrix. Taken together, these contrasts show that fragmentation in WGS-based AMR interpretation reflects not only database coverage but also differences in analytical function, taxon dependence, curation depth, and reporting logic. In practice, reproducible interpretation often requires coordinated use of complementary resources rather than reliance on a single database.

Organism-specific workflows remain important, particularly for pathogens such as *M. tuberculosis*, where resistance interpretation depends heavily on curated mutation catalogues, standardized genomic drug-susceptibility-testing workflows, and phenotype-linked validation (Roychowdhury et al., 2015; Gygli et al., 2019; Al-Ouqaili, 2022). Similar confirm-then-sequence models are used in foodborne surveillance systems for Salmonella, Shiga toxin-producing *E. coli* (STEC), and Listeria (Ribot et al., 2019), as well as in gonococcal surveillance for Neisseria gonorrhoeae (Unemo et al., 2019), illustrating how organism-specific characterization remains important within broader efforts toward harmonized genomic AMR surveillance (Supplementary Table 5). Therefore, harmonized AMR surveillance could preserve organism-specific interpretation rules while improving cross-resource reporting consistency.

Overall, the reproducibility of WGS-based AMR inference depends on database curation depth, update frequency, ontology alignment, metadata quality, analytical thresholds, and external benchmarking. In practice, laboratories often combine complementary resources rather than relying on a single database. For example, AMRFinderPlus, ResFinder/PointFinder, CARD/RGI, SRST2, ARIBA, and ABRicate support different isolate-based AMR detection strategies, whereas PlasmidFinder, MOB-suite-like workflows, and long-read or hybrid assemblies can add plasmid and MGE context where required. Broader platforms such as NDARO and BV-BRC (Bacterial and Viral Bioinformatics Resource Center; incorporating the former bacterial PATRIC resource) support surveillance metadata, standardized vocabularies, and comparative pathogen genomics, while MEGARes, SARG, and BacMet extend analysis toward environmental, metagenomic, and One Health resistome applications. These resources are therefore best interpreted as complementary modules within reproducible, version-controlled AMR surveillance workflows rather than interchangeable databases. Additional sector-specific surveillance applications of selected AMR resources are summarized in Supplementary Table 7.

## 4 Harmonizing WGS-based AMR surveillance for global monitoring

The worldwide spread of AMR transcends national borders and increasingly necessitates genomic surveillance systems that facilitate the early identification of emerging threats and coordinated responses in public health. WGS has enhanced AMR monitoring through the prompt detection of resistance determinants and high-resolution transmission tracking. Nonetheless, the lack of standardized analytical protocols, metadata structures, and reporting practices persists as a barrier to comparability among laboratories and hampers international collaboration. Reproducible surveillance, therefore, depends not only on sequencing capacity but also on standardized workflows, consistent metadata, and transparent provenance, including explicit reporting of software versions, database versions, and analytical thresholds. The molecular identification markers relevant to AMR/WGS workflows are detailed in Supplementary Table 8, while their placement within the broader progression from molecular identification and genomic resistance profiling to repository integration and surveillance analytics is illustrated in Supplementary Figure 1.

Within this landscape, harmonization should not be equated with the creation of a single universal AMR database that replaces specialized resources. A more realistic approach is federated harmonization, in which databases and analytical tools retain their distinct strengths while adopting shared identifiers, transparent evidence grading, version-controlled crosswalks, common reporting fields, and agreed quality-control metadata. Such a model would require sustainable governance, long-term funding, auditing of database updates, and mechanisms for resolving conflicting genotype–phenotype interpretations across resources. In clinical contexts, harmonized outputs would additionally require clinical validation, reproducibility standards, and phenotypic benchmarking before being used to inform direct therapeutic decision-making.

Several international initiatives provide valuable foundations for more harmonized genomic AMR surveillance. WHO Global Antimicrobial Resistance and Use Surveillance System (GLASS) offers a well-established framework for the standardized collection, analysis, interpretation, and sharing of AMR data, while supporting the progressive integration of One Health surveillance. Complementing this, the WHO Global Genomic Surveillance Strategy further promotes interoperable genomic-surveillance systems, responsible data exchange, and connectivity across surveillance networks (World Health Organization [WHO], 2020, 2022, 2025). At the interpretation level, EUCAST provides guidance relevant to the integration of WGS-derived evidence with phenotypic AST and breakpoint interpretation (Ellington et al., 2017), while its WGS–AST Subcommittee evaluates genomic prediction frameworks (Samuelsen et al., 2026). Complementary laboratory standards, including Clinical and Laboratory Standards Institute (CLSI) guidance for the analysis and presentation of cumulative antimicrobial susceptibility data, can support more consistent phenotype-based surveillance reporting and antibiogram development; however, they do not by themselves establish interoperability across WGS-AMR databases or genomic prediction workflows (Clinical and Laboratory Standards Institute [CLSI], 2022). Additionally, pathogen-specific networks such as PulseNet demonstrate the effectiveness of standardized genomic protocols and coordinated data sharing in outbreak detection and foodborne disease surveillance (Ribot et al., 2019). Taken together, these initiatives operate through distinct governance structures, evidence models, database scopes, and reporting conventions, supporting the need for federated harmonization (Perestrelo et al., 2023; Djordjevic et al., 2024).

These governance and standardization initiatives should be distinguished from complementary AMR-resource ecosystems. NCBI Pathogen Detection and NDARO (National Center for Biotechnology Information, 2023), CARD (Alcock et al., 2023), ResFinder and PointFinder (Zankari et al., 2017; Bortolaia et al., 2020), and BV-BRC/PATRIC (Wattam et al., 2017; Shukla et al., 2026) provide complementary resources for AMR determinant curation, resistance interpretation, surveillance-linked data integration, and comparative pathogen genomics. In parallel, One Health initiatives and international strain-level data-sharing efforts emphasize common metadata, reproducible workflows, and cross-sector comparability across human, animal, food-chain, and environmental domains (Queenan et al., 2016; Kahn, 2017; Perestrelo et al., 2023; Djordjevic et al., 2024). Together, these efforts provide an important foundation for harmonization; however, they do not yet establish a universally adopted cross-database AMR ontology, version-controlled crosswalk system, evidence-grading model, or provenance-aware reporting schema. This remaining gap supports federated harmonization rather than reliance on a single universal database (Clausen et al., 2016; Papp and Solymosi, 2022; Djordjevic et al., 2024).

Federated harmonization, however, presents substantial practical and methodological challenges. Effective interoperability would require standardized curation criteria, stable identifiers, transparent evidence grading, robust version control, auditable update histories, and long-term coordination across database-hosting organizations. Sustaining this coordination may be difficult without dedicated funding, shared maintenance responsibilities, and regular external quality assessment. A further challenge is that genotype-based resistance predictions require rigorous linkage to phenotypic AST or MIC data, which is resource-intensive and may not be feasible across all organisms, drug classes, and contributing institutions. Even established databases may include determinants whose phenotypic effects are context-dependent or incompletely validated. Therefore, harmonized database outputs should not automatically be interpreted as clinically actionable resistance calls. For example, some efflux-associated determinants may be associated with modest MIC shifts rather than categorical clinical resistance. Such outputs can support surveillance, benchmarking, and secondary analyses, whereas direct clinical decision-making requires stricter validation, reproducibility standards, and phenotype-linked interpretation frameworks (Burnham et al., 2017; Ellington et al., 2017; Rebelo et al., 2022).

Comparative evaluations show that major AMR resources provide complementary strengths rather than interchangeable outputs, as summarized in Table 2 and Supplementary Table 7. CARD, for example, is mechanism-oriented and frequently updated, which can improve the interpretability of resistance pathways, although representation of newly described or rare determinants may still lag behind rapid literature emergence (Jia et al., 2017; Alcock et al., 2020, 2023). ResFinder is commonly used for acquired resistance gene detection, but as a similarity-based screening approach, it provides limited structural information on plasmid context unless paired with plasmid-focused resources such as PlasmidFinder and supported by long-read or hybrid assemblies for improved MGE resolution (Carattoli et al., 2014; Maboni et al., 2022). ARGminer has the potential to expand representation through crowdsourced curation, encompassing environmentally derived genes. However, the variability in user-contributed submissions may impact both consistency and completeness (Arango-Argoty et al., 2020; Maboni et al., 2022). NDARO provides broad coverage of resistance genes associated with human pathogens, yet its emphasis on clinical pathogens may limit representation of environmental and animal reservoirs in some One Health applications (National Center for Biotechnology Information, 2023). Because discordant AMR predictions remain common across databases and tools, predictions with major clinical or surveillance implications should be reported with explicit provenance, threshold settings, and versioning, and corroborated with orthogonal or phenotypic evidence where feasible.

A major obstacle to comparability is that AMR databases and analytical pipelines do not represent or interpret all resistance determinants in the same way. Differences in reference content, gene-family definitions, curation policies, identity and coverage thresholds, input data, and software or database versions can cause the same genome to yield non-identical AMR calls across workflows, particularly for partial, mosaic, or closely related alleles (Clausen et al., 2016; Tagini and Greub, 2017; Papp and Solymosi, 2022). Comparative studies have shown that AMR-gene detection may vary across annotation platforms and sequencing-data types, reinforcing that database choice and analytical configuration can materially influence surveillance outputs (Clausen et al., 2016; Tagini and Greub, 2017). These discrepancies highlight the need for shared reference panels, transparent version reporting, standardized benchmark datasets, and interlaboratory evaluation of analytical workflows (Clausen et al., 2016; Doyle et al., 2020). In routine surveillance, outputs should therefore be accompanied by database and software versions, decision thresholds, and explicit statements of uncertainty and genomic context (Clausen et al., 2016; Ellington et al., 2017; Papp and Solymosi, 2022).

Resource constraints in low- and middle-income countries continue to be a significant obstacle to regular WGS-based AMR surveillance. Limited computational infrastructure, storage, bandwidth, and bioinformatics expertise can hinder implementation even when sequencing platforms are accessible (Kwong et al., 2015; Price et al., 2023; Djordjevic et al., 2024). Portable sequencing devices and cloud- or web-based analysis environments may reduce some of these barriers and support more standardized deployment across sites (Rowe et al., 2015). However, successful implementation still depends on reliable connectivity, sustainable financing, data governance and privacy frameworks, workforce training, and standardized version-controlled pipelines supported by external quality assessment. A pragmatic model is a hybrid implementation architecture in which basecalling and initial QC are performed locally, followed by secure cloud execution of compute-intensive analytical steps under standardized operating procedures.

Overall, global AMR surveillance would benefit from a more consistent framework for AMR gene annotation, genotype–phenotype interpretation, database versioning, and across-resource reporting (Clausen et al., 2016; Burnham et al., 2017; Ellington et al., 2017; Papp and Solymosi, 2022; Rebelo et al., 2022; Djordjevic et al., 2024). Persistent differences in gene-family boundaries, allele nomenclature, evidence models, and resistance rules continue to drive fragmented inference. Progress will therefore require shared ontologies; standardized reporting fields, including identity and coverage thresholds, genomic context, and evidence strength; as well as machine-readable schemas and interoperable application programming interfaces for data and metadata exchange. These efforts should be paired with public benchmark panels, external quality assessments, and transparent versioning practices. Rather than replacing specialized databases, a federated and centrally indexed model could support harmonized analytics while preserving local control and context-specific strengths. Such governance should also promote equitable participation, including laboratories in resource-limited settings, to strengthen both global surveillance and downstream clinical decision support.

## 5 Pathways to precision: toward global standards in phenotypic and genotypic AMR testing

Although WGS has substantial potential as a diagnostic and surveillance tool, particularly in hospital and public-health settings (Ellington et al., 2017; Djordjevic et al., 2024), the relationship between genotype and phenotype remains biologically and methodologically complex. Detection of resistance genes or mutations does not always translate directly into measured phenotypic resistance. The predictive value of genomic data, therefore, depends on interpretation within biological context, standardized analytical frameworks, and, where clinical action is intended, validation against phenotypic AST.

Representative discordance patterns illustrate why detection of a resistance determinant should not automatically be equated with categorical phenotypic resistance (Burnham et al., 2017; Ellington et al., 2017; Rose et al., 2023). For example, an acquired β-lactamase gene may be present without categorical phenotypic resistance when the allele is truncated or otherwise non-functional, or when its expression or copy number is insufficient to raise the MIC above the applicable breakpoint (Burnham et al., 2017; Ellington et al., 2017; Silva and Khare, 2024). Conversely, phenotypic resistance may occur despite the absence of a recognizable acquired resistance gene because resistance can be mediated by chromosomal target mutations, reduced permeability through porin loss, efflux upregulation, gene amplification, or other regulatory mechanisms (Burnham et al., 2017; Jeukens et al., 2017; Choi and Lee, 2019; Nicoloff et al., 2019; Silva and Khare, 2024; Wu et al., 2024). In *Pseudomonas aeruginosa*, reduced OprD-mediated permeability can contribute to carbapenem resistance, whereas altered efflux activity may influence susceptibility across multiple antimicrobial classes (Jeukens et al., 2017; Wu et al., 2024). In Enterobacterales, copy-number variation and amplification of β-lactamase-bearing elements can alter MICs and complicate interpretation based only on a simple gene-presence call, particularly in KPC-associated resistance phenotypes (Nicoloff et al., 2019; Di Pilato et al., 2023; Lipworth et al., 2024; Silva and Khare, 2024). These examples reinforce the need to interpret genomic findings alongside standardized phenotypic AST or MIC data, particularly for clinically consequential resistance predictions (Burnham et al., 2017; Ellington et al., 2017; Feldgarden et al., 2019; Rebelo et al., 2022). Beyond these representative examples, phenotypic variation may also reflect biofilm-associated tolerance, inoculum effects, and other physiological states that can shift MIC values without a corresponding change in the core resistance genotype (Lenhard and Bulman, 2019; Yan and Bassler, 2019). These factors reinforce the need to interpret genomic predictions within their biological and methodological context rather than as stand-alone categorical resistance calls.

Methodological variability further complicates genotype–phenotype interpretation. Differences in AST platforms, breakpoint systems, laboratory reproducibility, and reporting standards can all contribute to observed discordance between genomic prediction and measured susceptibility. For this reason, expert guidance, including EUCAST recommendations on WGS and AST integration, supports calibrated genomic interpretation alongside standardized phenotypic testing for high-consequence decisions (Ellington et al., 2017). Large benchmarking efforts, including AMRFinder evaluations and CRyPTIC analyses for first-line tuberculosis drugs, have shown strong agreement in selected organism–drug settings, but they also reinforce that performance remains context-specific rather than universally transferable (CRyPTIC Consortium and the 100, 000 Genomes Project, 2018; Feldgarden et al., 2019).

Benchmarking WGS-based AMR prediction, therefore, requires reference standards that reflect both microbiological and clinical perspectives. EUCAST recommends epidemiological cut-off values (ECOFFs) as the primary microbiological reference, with clinical breakpoints used to provide therapeutic context (Ellington et al., 2017). Clinical breakpoints integrate microbiological distributions with pharmacokinetic and pharmacodynamic parameters, dosing regimens, and infection-site considerations. However, their exclusive use in WGS validation is limited by differences between interpretive systems such as EUCAST and CLSI, the absence of breakpoints for some uncommon organism–drug combinations, and the possibility of overlooking emerging resistance when MICs remain just below a clinical threshold despite the presence of non-wild-type mechanisms (Ellington et al., 2017; Gygli et al., 2019; Simner et al., 2022). ECOFFs, in contrast, are derived from aggregate MIC distributions and distinguish wild-type from non-wild-type populations independently of treatment context (Kahlmeter and Turnidge, 2023). This improves reproducibility across studies, facilitates early recognition of resistance shifts, including small MIC increases caused by single-step mutations (Maganga et al., 2023), and reduces circularity in genotype–phenotype association. A dual-layer framework is therefore preferable, using ECOFFs as the microbiological benchmark and clinical breakpoints to interpret therapeutic significance.

Additional biological and technical sources of variability must also be acknowledged. Factors such as strain diversity, polygenic resistance architectures, pleiotropy, recombination, and environment-dependent expression can influence observed resistance. Furthermore, clinical outcomes are additionally shaped by host factors, polymicrobial interactions, conditions at the site of infection, and tissue-level pharmacokinetic and pharmacodynamic effects (Burnham et al., 2017; Shelburne et al., 2017). At the analytical level, sample quality, sequencing platform heterogeneity, incomplete reference databases, and inconsistent curation rules introduce further uncertainty (Clausen et al., 2016; Ellington et al., 2017; Papp and Solymosi, 2022). Improving agreement between computational prediction and empirical susceptibility, therefore, requires species–drug-specific curation, transparent decision rules, versioned AMR databases, and reporting frameworks that distinguish strong, moderate, and uncertain evidence (Rebelo et al., 2022).

Machine-learning approaches may help improve predictive concordance by integrating broader genomic feature sets into susceptibility classification. Tools such as AMR-Diag illustrate this potential, but performance remains dependent on training-data completeness, phenotype quality, breakpoint harmonization, sequencing variability, database update frequency, and genomic context (Avershina et al., 2021; Sakagianni et al., 2023; Li et al., 2024; Pennisi et al., 2025). Before such models are used in clinical or high-stakes surveillance settings, they should undergo prospective multisite validation and be implemented within version-controlled pipelines that provide calibrated probability outputs and explicit uncertainty reporting. Ongoing governance is also needed to monitor model drift, document failure modes, and define circumstances requiring orthogonal confirmation or phenotypic verification (Sakagianni et al., 2023; Li et al., 2024; Pennisi et al., 2025).

To convert WGS-based predictions into standardized and clinically meaningful reports, workflows should incorporate predefined quality checks, transparent documentation of software and database versions, clear reporting of analytical parameters, and appropriate validation steps before reporting. A tiered analytical framework can integrate QC, typing, gene and variant calling, MGE analysis, phylogenomic context, and adjudication of genotype–phenotype relationships, supported by auditable logs and interoperable outputs based on machine-readable schemas and standardized ontologies. When high-consequence calls are made, independent confirmation and/or phenotypic verification should remain part of the reporting pathway (Ellington et al., 2017; Feldgarden et al., 2019; Rebelo et al., 2022; Rose et al., 2023).

Genome editing tools such as CRISPR–Cas systems can also support this evidence framework in research settings by enabling targeted disruption or confirmation of candidate resistance determinants, thereby strengthening causal interpretation of genotype–phenotype associations (Jwair et al., 2023; Al-Ouqaili et al., 2025). In this context, CRISPR–Cas should be viewed primarily as a functional validation approach rather than a routine diagnostic component, but it may complement WGS-based prediction by reinforcing mechanistic evidence for specific loci.

Overall, progress toward precision AMR prediction requires harmonized AST practices, curated and versioned reference databases, transparent reporting of uncertainty, and coordinated external validation frameworks. Together, these elements can improve cross-study comparability, strengthen surveillance interpretation, and support more clinically meaningful translation of WGS-based AMR outputs (Burnham et al., 2017; Ellington et al., 2017; CRyPTIC Consortium and the 100, 000 Genomes Project, 2018; Feldgarden et al., 2019; Rebelo et al., 2022).

### 5.1 Barriers to routine clinical implementation of WGS-based AMR prediction

Despite its value for surveillance and outbreak investigation, routine clinical implementation of WGS-based AMR prediction remains limited by practical barriers, including sequencing turnaround time, capital and consumable costs, laboratory workflow integration, specimen and culture requirements, bioinformatics infrastructure, data storage, and workforce capacity (Kwong et al., 2015; Quainoo et al., 2017; Price et al., 2023). Additional challenges include heterogeneity in AMR database content, tool performance, analytical thresholds, software and database version control, and the need for quality-assured validation against phenotypic AST (Clausen et al., 2016; Feldgarden et al., 2019; Doyle et al., 2020; Papp and Solymosi, 2022). Clinical implementation is particularly challenging for organism–drug combinations with complex, polygenic, regulation-dependent, or incompletely characterized resistance mechanisms, where genomic detection of genes or variants may not reliably predict the measured phenotype; these limitations are illustrated in *P. aeruginosa* (Jeukens et al., 2017; Wu et al., 2024). Regulatory requirements, inconsistent breakpoint interpretation, limited availability of standardized external quality-assessment approaches, and uncertainty regarding responsibility for interpretation and reporting also remain important obstacles. Therefore, WGS currently provides its greatest value as a complementary approach for surveillance, outbreak investigation, resistance-mechanism characterization, and selected validated organism–drug predictions, rather than as a universal replacement for conventional AST (Ellington et al., 2017; Quainoo et al., 2017; Feldgarden et al., 2019; Rebelo et al., 2022).

## 6 Limitations of WGS-based AMR inference

Despite major advances in sequencing technologies and AMR prediction tools, WGS-based AMR inference has important limitations that should be explicitly recognized in clinical and surveillance contexts. First, as detailed in section 5, detection of an AMR gene, allele, or resistance-associated mutation does not automatically establish categorical phenotypic resistance. Resistance expression may depend on promoter activity, gene regulation, copy number, porin loss, efflux activity, plasmid dosage, gene truncation, allele functionality, and environmental conditions (Burnham et al., 2017; Ellington et al., 2017; Jeukens et al., 2017; Choi and Lee, 2019; Nicoloff et al., 2019; Silva and Khare, 2024; Wu et al., 2024). Conversely, phenotypic resistance may occur through mechanisms that are incompletely represented in current databases or are difficult to infer from sequence data alone (Jeukens et al., 2017; Rebelo et al., 2022).

Second, AMR outputs are strongly shaped by database scope, nomenclature, curation rules, update timing, identity and coverage thresholds, and software implementation (Alcock et al., 2020; Papp and Solymosi, 2022). The same genome can produce different results across tools because resources differ in how they define gene families, classify alleles, include partial genes, treat mosaic sequences, and link genotypes to antimicrobial classes. These differences are especially important for uncommon organism–drug combinations, novel alleles, incomplete genes, multicopy loci, and mutation-mediated resistance (Alcock et al., 2020; Bortolaia et al., 2020; Feldgarden et al., 2021; Papp and Solymosi, 2022). Therefore, WGS-AMR reporting should include software versions, database versions, thresholds, input type, sequencing platform, and quality-control criteria (Clausen et al., 2016; Ellington et al., 2017; Papp and Solymosi, 2022).

Third, short-read sequencing can detect many acquired ARGs and small variants, but it may struggle to resolve multicopy genes, mixed allelic populations, repetitive regions, plasmids, transposons, and insertion-sequence-associated resistance loci (Carattoli et al., 2014; Arredondo-Alonso et al., 2017). These limitations can affect genotype–phenotype interpretation, particularly when resistance depends on the number of mutated gene copies or the structural context of an AMR determinant (Arredondo-Alonso et al., 2017). Long-read and hybrid workflows can improve the resolution of structural analysis, but require meticulous oversight of version control, polishing strategies, coverage assessment, and validation procedures (Carattoli et al., 2014; Wick et al., 2021, 2023).

Fourth, high-stakes clinical implementation requires expert interpretation and validation against phenotypic AST, especially MIC-based testing, for decisions of high consequence (Burnham et al., 2017; Ellington et al., 2017; Rebelo et al., 2022). Genomic predictions are most reliable when supported by curated organism-specific interpretation frameworks, benchmarked pipelines, external quality assessment, and transparent uncertainty reporting (Feldgarden et al., 2019; Rebelo et al., 2022). For these reasons, phenotypic AST remains the clinical reference standard in many settings, while WGS is best understood as a powerful complementary tool for mechanism detection, surveillance, outbreak analysis, and contextual AMR interpretation (Burnham et al., 2017; Ellington et al., 2017; Rebelo et al., 2022).

## 7 Integrating artificial intelligence and machine learning into WGS-based AMR analysis

Artificial intelligence (AI) and machine learning (ML) approaches may support WGS-based AMR analysis by prioritizing candidate resistance determinants, identifying complex genomic patterns, and improving prediction in datasets where simple identity-based matching is insufficient (Arango-Argoty et al., 2018; Sakagianni et al., 2023; Li et al., 2024; Pennisi et al., 2025). However, these approaches should be interpreted as complementary analytical layers rather than replacements for curated AMR databases, validated rule-based pipelines, and phenotypic AST when clinical interpretation is required. Their performance is highly dependent on training-data composition, organism representation, phenotype quality, breakpoint definitions, sequencing platform, and external validation (Arango-Argoty et al., 2018; Sakagianni et al., 2023; Li et al., 2024; Pennisi et al., 2025). In practice, tools such as DeepARG are most relevant as complementary approaches for metagenomic or discovery-oriented resistome analysis, whereas clinical or high-stakes isolate interpretation still requires curated databases, transparent decision rules, version-controlled workflows, and phenotypic or orthogonal validation where appropriate (Ellington et al., 2017; Feldgarden et al., 2019; Rebelo et al., 2022).

Deep-learning approaches may extend AMR prediction beyond direct sequence-identity matching by learning patterns from curated resistance-gene datasets and broader genomic features. However, their outputs remain model-dependent and should be interpreted as probabilistic or prioritization evidence rather than definitive clinical resistance calls (Arango-Argoty et al., 2018; Sakagianni et al., 2023; Li et al., 2024; Pennisi et al., 2025). Nevertheless, broader clinical or public health implementation remains limited by training-data representativeness, reproducibility across sites, and algorithmic opacity. Without calibrated probabilities, transparent model provenance, and explicit documentation of input data, model version, and update history, AI/ML predictions are difficult to audit and remain vulnerable to bias, shortcut learning, and domain shift (Sakagianni et al., 2023; Li et al., 2024; Pennisi et al., 2025). Future development should therefore prioritize standardized reference datasets, multisite external validation, uncertainty-calibrated reporting, and ongoing monitoring for model drift. In practical terms, existing curated bioinformatics frameworks should remain the primary inference layer, with AI/ML serving as a complementary and externally validated component rather than a replacement (Ellington et al., 2017; Jeukens et al., 2017; Feldgarden et al., 2019; Rebelo et al., 2022).

Whole-genome sequencing-based prediction also has inherent biological limits that AI/ML alone cannot solve. Sequence-based workflows do not directly capture transient tolerance phenotypes, epigenetic effects, or adaptive states such as persistence and biofilm-associated tolerance. More context-sensitive interpretation may therefore require integrated approaches that combine genomic prediction with targeted phenotypic, transcriptomic, or functional assays, as relevant (Burnham et al., 2017; Hall and Mah, 2017; Jeukens et al., 2017; Rebelo et al., 2022). In this setting, AI and ML are best viewed as augmentative analytical layers that may improve prioritization and pattern recognition, but only when embedded within reproducible, interpretable, and biologically grounded workflows.

## 8 AMR-GenoLink as a framework for harmonized AMR surveillance

Antimicrobial resistance-GenoLink is proposed here as a conceptual reference architecture rather than an established database, deployed platform, or replacement for existing AMR tools. Its role is to preserve the distinction between detected genomic features, interpreted resistance inferences, phenotype-linked validation, and final evidence-tiered reporting. It illustrates how outputs from validated AMR resources could be organized through provenance-aware and interoperable reporting across One Health surveillance settings (Djordjevic et al., 2024). This framing addresses persistent fragmentation across AMR databases, inconsistent annotation practices, and limited interoperability across human, animal, and environmental sectors. As emphasized throughout this review, the major challenge is no longer simply generating more WGS data, but ensuring that outputs from different resources and laboratories can be interpreted consistently and reported transparently.

Unlike existing AMR databases or surveillance platforms, AMR-GenoLink is not proposed as a new resistance-gene repository, a replacement for CARD, ResFinder, AMRFinderPlus, NDARO, or BV-BRC, or a stand-alone diagnostic platform. Instead, it is a conceptual interoperability layer designed to connect outputs from curated resources through common metadata fields, evidence tiers, version-controlled crosswalks, and provenance-aware reporting. Its primary purpose is therefore to improve comparability between laboratories, databases, and One Health sectors while preserving the specialized functions of existing resources. Accordingly, AMR-GenoLink does not independently generate resistance calls or replace phenotypic AST; rather, it provides a structured framework for integrating, documenting, grading, and reporting evidence generated by validated tools and reference resources. For clinical or applied use, AMR-GenoLink should not integrate all available AMR databases indiscriminately. Instead, the framework should prioritize well-curated, experimentally supported, version-controlled, and benchmarked reference resources, particularly when outputs are intended for diagnostic use or high-stakes surveillance interpretation. Reference resources should also be stratified according to data type and analytical purposes. Isolate-based AMR interpretation should be separated from metagenomic resistome profiling because single-colony WGS and mixed-community sequencing require different assumptions, thresholds, abundance models, contamination controls, and reporting standards. Similarly, curated chromosomal mutation catalogues should be distinguished from acquired AMR gene reference sets because mutation-mediated resistance and gene presence/absence profiling represent different biological mechanisms and generate different levels of interpretive evidence. For clinical applicability, AMR-GenoLink would require structured links between genotypic outputs and phenotypic AST or MIC data, transparent evidence grading, version-controlled database crosswalks, auditable update histories, and validation frameworks that distinguish surveillance signals from clinically actionable resistance predictions. Discovery-oriented resources and AI/ML components may support horizon scanning and signal prioritization, but should remain optional modules subject to the validation, uncertainty-reporting, transparency, and version-control requirements outlined in section 7.

The proposed AMR-GenoLink framework is intended to illustrate how WGS-derived AMR evidence from multiple sources could be consolidated and interpreted while emphasizing standardized data handling, consistent annotation of resistance determinants (Figure 3), and transparent provenance, including database versions, software versions, and threshold settings (Djordjevic et al., 2024). The proposed central interoperability layer would operationalize this concept through metadata standards, version-controlled crosswalks, evidence grading, provenance tracking, and reporting templates. Its core components include interoperable data ingestion that standardizes metadata across human, animal, and environmental source domains; selective database-supported resistome reporting that links genes, alleles, chromosomal mutations, plasmid/MGE context, and phenotypic AST or MIC data to standardized ontologies and evidence strength; separate analytical pathways for isolate-based WGS and metagenomic resistome data; and optional analytical modules that can support prioritization, trend monitoring, and signal detection, including AI/ML approaches where these have been appropriately validated. By linking genomic findings to consistent reporting fields and structural context, including plasmid or MGE association where available, the framework could support standardized AMR reporting and surveillance alerting while also enabling cross-sector comparison under a One Health model.

**FIGURE 3:**
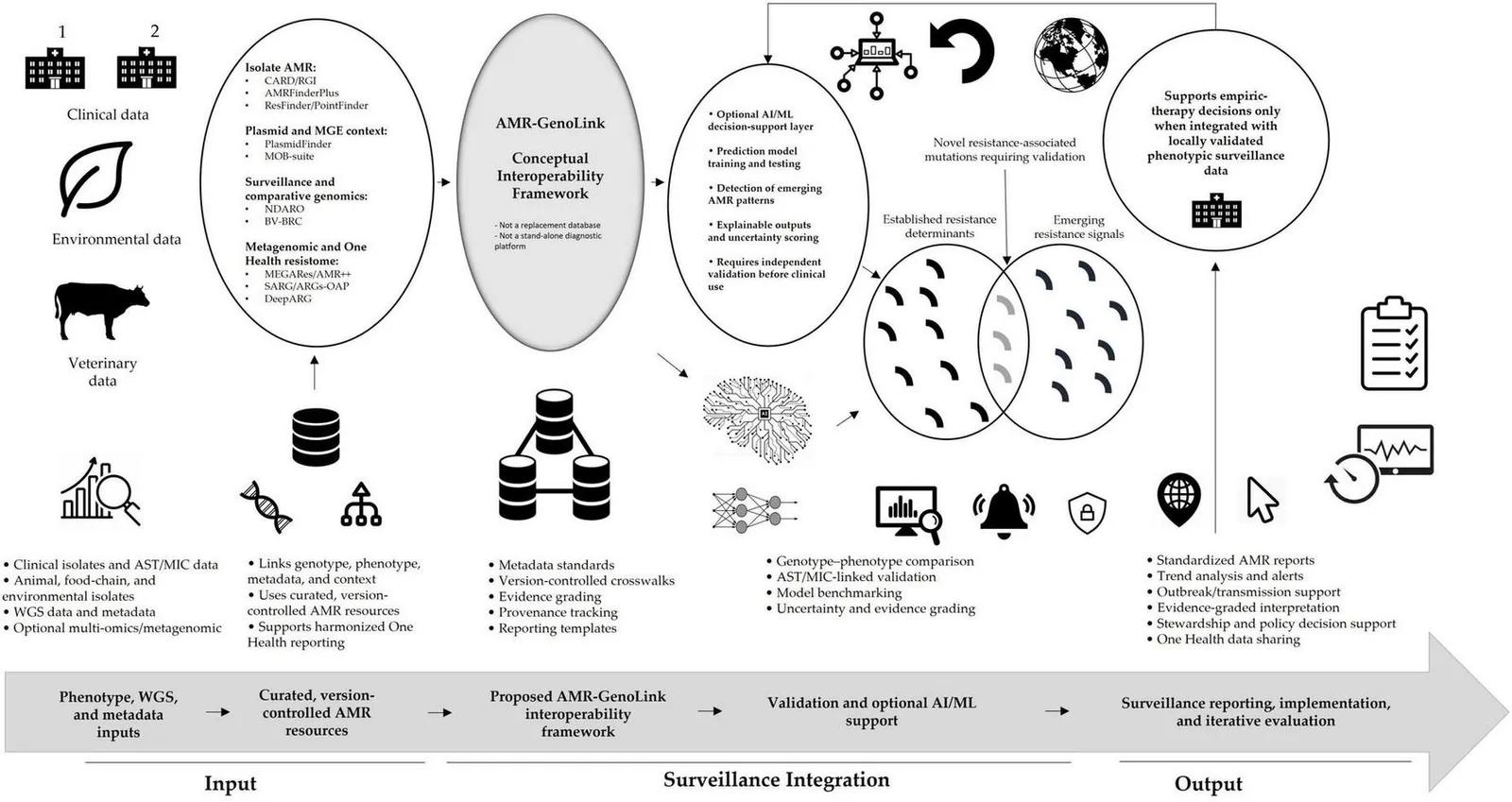
Proposed antimicrobial resistance (AMR)-GenoLink interoperability framework for harmonized whole-genome sequencing (WGS)-based AMR surveillance and reporting. The framework integrates phenotypic data, isolate WGS or metagenomic outputs, contextual metadata, and curated AMR resources through a central interoperability layer comprising metadata standards, version-controlled crosswalks, evidence grading, provenance tracking, and reporting templates. AMR-GenoLink is presented as a conceptual reference architecture rather than an established platform, replacement AMR database, or stand-alone diagnostic system. The framework supports genotype–phenotype comparison, plasmid/MGE contextualization, evidence grading, uncertainty reporting, and One Health surveillance across human, animal, food-chain, and environmental sectors. AI/ML components are shown as optional decision-support layers that require benchmarking, explainability, and independent validation before clinical or public-health implementation. Outputs include standardized AMR reports, surveillance alerts, and audit-ready reporting across participating sites.

Implementation should be phased and flexible so that the framework can accommodate evolving standards and sites with different data structures and levels of technical capacity. A fundamental prerequisite is interoperability, encompassing machine-readable schemas, stable identifiers, and controlled vocabularies that facilitate the comparison of outputs across laboratories and over time. AI/ML modules may aid in prioritizing signals or identifying annotation inconsistencies; however, they should be used only within validated, uncertainty-calibrated reporting and version-controlled workflows to ensure auditability and mitigate model drift. Overall, AMR-GenoLink is best understood as a proposed harmonization framework centered on data standards, reproducible workflows, and provenance-aware reporting for scalable One Health AMR surveillance.

## 9 Conclusion and future perspectives

Whole-genome sequencing, supported by advances in computational analysis, is strengthening AMR surveillance by enabling high-resolution detection of resistance determinants, transmission inference, and, when assemblies permit, interpretation of resistance genes within their genomic context, including MGEs. However, the practical value of these advances depends less on any single sequencing platform or analytical tool than on reproducible workflows, transparent benchmarking, and context-aware interpretation that can be compared across laboratories and sectors.

Decision-ready interpretation remains limited by both technical and biological constraints. Short-read data often fragment repeat-rich and mobilome-associated structures, whereas long-read workflows remain sensitive to chemistry, basecalling, polishing, and software-version effects, particularly in low-complexity regions. Additional uncertainty arises from uneven coverage, contamination, barcode crosstalk, reference bias in mapping-based workflows, and chimeric structures introduced during assembly. These limitations become especially important when surveillance depends on plasmid or transposon reconstruction, copy-number interpretation, low-frequency subpopulation detection, or when genotype–phenotype concordance is weakened by recombination, epistasis, regulation, mixed infection, or environment-dependent expression.

Three priorities emerge from this review. First, AMR ontologies and reporting fields should be standardized using stable identifiers, explicit evidence grading, and machine-readable data structures. Second, shared reference materials and open benchmark datasets should be expanded across short-read, long-read, hybrid, and metagenomic workflows, supported by external quality assessment and containerized, version-controlled pipelines. Third, predictive models should be implemented with calibrated uncertainty reporting and monitored over time, while retaining interpretable mechanism-aware rules for high-stakes applications. Progress should be judged not only by analytical sophistication, but also by detection performance, reproducibility across sites, concordance with phenotypic testing, and operational feasibility.

Within this landscape, AMR-GenoLink is proposed as a conceptual reference framework for interoperable ingestion, standardized reporting, and integration of WGS-derived AMR evidence across clinical, veterinary, and environmental domains. Progress will depend on coordinated database alignment, robust metadata standards, and practical interoperability agreements that support common schemas, application programming interfaces, and synchronized versioned releases. Together, these steps can convert heterogeneous genomic findings into comparable, auditable, and actionable AMR evidence by distinguishing what was detected, how it was interpreted, whether it was validated, and what level of reporting is justified. This approach can support surveillance, infection control, antimicrobial stewardship, and One Health decision-making.

## 10 Methodological approach

This article is a narrative review supported by a structured literature-identification approach; it was not designed as a systematic review or meta-analysis. It synthesizes current evidence on bacterial WGS workflows, AMR interpretation tools, and database harmonization relevant to genomic surveillance. A focused search was performed in PubMed, Web of Science, and Google Scholar using combinations of terms such as “next-generation sequencing,” “whole-genome sequencing,” “bacterial genomic tools,” “bioinformatics platforms,” “bacterial genomic data analysis,” “genomic data quality control,” “genome assembly tools,” “AMR databases,” and “genome annotation tools.” Included studies were peer-reviewed articles published in English and comprised both original research and relevant review papers addressing the development, application, or comparison of genomic tools in healthcare and research settings. Non-genomic studies and non-research-based sources were excluded from the evidence synthesis. Official software documentation, vendor pages, and database websites were consulted only to verify current tool names, access routes, input requirements, version-related details, and database availability; they were not used as primary evidence for scientific performance claims. References cited in key articles were additionally screened to identify further relevant publications. The final body of literature was organized into thematic sections based on AMR detection, genotype–phenotype interpretation, database fragmentation, tool interoperability, validation, and harmonization needs. A comparative synthesis was then undertaken to assess the strengths, limitations, and interoperability of these resources, with particular emphasis on areas where harmonization across tools and databases remains necessary. Relevant database websites are provided in Supplementary Table 9.
