# Supplementary material for: Interpreting antimicrobial resistance from bacterial whole-genome sequencing: prediction tools, database fragmentation, analytical trade-offs, and harmonized reporting
Source: Front Microbiol. 2026 Sep 14;17:1849165. doi: 10.3389/fmicb.2026.1849165 (PMC13617433; doi:10.3389/fmicb.2026.1849165)
Supplement: Supplementary file 1 [file Table_1.docx]

Supplementary Material

| **Item** | **Title** |
| --- | --- |
| **Table S1** | Comparison of data quality-control and preprocessing tools for sequencing workflows |
| **Table S2** | Performance comparison of short-read bacterial genome assembly tools used in benchmarking datasets |
| **Table S3** | Long-read assembly tools and ONT-linked workflows relevant to AMR genomic-context interpretation |
| **Table S4** | Hybrid assembly workflows relevant to AMR genomic-context interpretation |
| **Table S5** | Illustrative taxon-linked applicability of selected AMR resources across representative clinical and One Health taxa |
| **Table S6** | Genome-analysis tools relevant to WGS-AMR workflows, including alignment, basecalling, polishing, variant calling, contamination screening, visualization, phylogenetic context, and mobile genetic element analysis |
| **Table S7** | Practical scope and surveillance use of selected AMR resources across human, animal, and environmental sectors |
| **Table S8** | Molecular identification markers and their relevance to AMR/WGS workflows |
| **Table S9** | Representative web resources for the AMR databases and platforms discussed in the manuscript |
| **Figure S1** | Workflow from molecular identification to database-supported AMR surveillance analysis |

**Supplementary Table S1:** Comparison of data quality control and preprocessing tools for sequencing workflows.

| **Software** | **Quality Check** | **Cleaning**  **/ Pre-**  **processing** | **Read type** | **Key features** | **Customization**  **Options** | **Ref.** |
| --- | --- | --- | --- | --- | --- | --- |
| **Raw-read QC / preprocessing** | | | | | | |
| **FastQC** | Yes | No | Short reads | - Per-base quality, GC content, sequence duplication, and adapter-content summaries support rapid assessment of read quality. - Provides rapid visual screening before downstream AMR analysis | Limited | [1] |
| **NGSQC Toolkit** | Yes | Yes |  | - Batch QC for legacy pipelines - Batch trimming, filtering, and adapter removal using customizable criteria | Advanced | [2] |
| **AfterQC** | Yes | Yes |  | - Automatic error correction, trimming, and artifact detection - One-command cleanup for cohort-scale datasets | Few | [3] |
| **Fastp^1^** | Yes | Yes |  | - Integrated quality filtering, trimming, and adapter removal - Generates summary QC reports | Moderate | [4,5] |
| **Cutadapt** | No | Yes |  | - Removes adapters and poly-A tails - Trims low-quality bases | Advanced | [6] |
| **Trimmomatic** | No | Yes |  | - Adapter trimming with flexible options and quality filtering - Widely used in legacy and current short-read pipelines | Highly customizable trimming steps | [7] |
| **NanoQC** | Yes | No | Long reads | - Quick quality control for Nanopore reads - Provides summary plots and interactive QC visualizations | Few | [8] |
| **LongQC** | Yes | No |  | - Specialized QC for long-read sequencing (PacBio, Nanopore) - Diagnostics for read length and quality distributions | Few | [9] |
| **NanoPlot /**  **NanoFilt** | No | Yes |  | - Provides run-level QC visualizations and read filtering by quality score and length | Few | [8] |
| **Porechop** | No | Yes |  | - Trims adapters and barcodes from Nanopore reads, including split or chimeric reads | Limited trimming flexibility | [10,11] |
| **Chopper ^1^** | Yes | Yes |  | - Filters reads by length, quality, and GC content, and supports head/tail and quality-based trimming | Minimum and maximum lengths; thresholds for quality and GC content; cropping from the head or tail. | [12] |
| **NanoComp** | Yes | No |  | - Compares multiple long-read datasets using read length, quality, and identity metrics - Generates comparative plots and HTML or PNG outputs | Supports selection of plot type, dataset labels, colors, output format, and run-specific input grouping | [12] |
| **SOAPnuke** | Yes | Yes |  | - Integrated QC–preprocessing workflow for adapter trimming, quality filtering, polyG handling, and support for multiple sequencing modules | Module-specific configuration of adapter, quality, length, and thread settings | [13] |
| **RabbitQCPlus** | Yes | Yes | Short and long reads | - Fast QC, trimming, adapter removal, and filtering for short- and long-read datasets | Moderate | [14] |
| **Contamination / taxonomic screening** | | | | | | |
| **ConFindr** | Yes | No |  | - Detects intra- and cross-species contamination in raw Illumina reads using rMLST **^2^** markers | Supports rMLST or cgMLST modes, database-path configuration, and multithreading | [15] |
| **Kraken2** | Yes | No |  | - Rapid taxonomic screening using exact k-mer matching - Useful for contamination screening and taxonomic validation | Custom database construction; configurable confidence thresholds and output formats | [16] |
| **Assembly-level QC** | | | | | | |
| **BUSCO ^*^** | Yes | No | Assembled genomes /  annotations | - Assesses genome assemblies and annotations using conserved single-copy orthologs to estimate completeness. | Supports lineage-specific analysis for phylogenetically informed completeness assessment | [17,18] |
| **CheckM2 ^*^** | Yes | No |  | - Estimates genome completeness and contamination using marker genes and machine-learning models - Useful for genome-quality assessment in assembled bacterial genomes | Configurable model selection, threading, output options, and low-memory mode | [19,20] |
| **QUAST/WebQUAST ^*^** | Yes | No |  | - Calculates contiguity and structural metrics, including N50, NA50, and misassembly profiles | Supports comparison of multiple assemblies and configurable output formats | [21] |

**^1^** For pre-polishing read processing where applicable.

**^2^** rMLST refers to ribosomal multi-locus sequence typing (ConFindr).

**^*^ Note:** These are assembly-level assessment tools and are not used for raw-read QC. CheckM/CheckM2 estimate microbial genome completeness and contamination, QUAST/WebQUAST assess contiguity and structural assembly metrics, and BUSCO is best interpreted as an optional marker-gene completeness indicator and should not be used as a substitute for microbial contamination assessment.

**Supplementary Table S2.** Performance comparison of short-read bacterial genome assembly tools used in benchmarking datasets.

| **Tool** | **Assembly quality metrics** | **Runtime**/ **computational profile** | **Key features** | **Main limitations** | **Typical bacterial applications** | **User**  **accessibility** | **Error**  **correction** | **Ref.** |
| --- | --- | --- | --- | --- | --- | --- | --- | --- |
| **Shovill** | Can produce compact bacterial draft assemblies in benchmarked isolate datasets; contiguity and misassembly rates remain dataset-dependent* | High usability due to automated workflow | Generally fast in routine bacterial isolate workflows | Performance may decline in more complex assemblies, including those requiring chromosome and plasmid reconstruction | Automated pipeline for routine bacterial isolate genome assembly | High usability due  to automated workflow | Basic error correction included | [22] |
| **SKESA** | Can generate conservative bacterial assemblies with low error rates in suitable short-read datasets; contiguity is dataset-dependent | Automated workflow with relatively accessible use | Generally fast, memory-conscious, and user-friendly for routine bacterial isolate assembly | May generate fragmented assemblies and is less suitable for assemblies needing better structural resolution | Bacterial genome assembly, especially small bacterial genomes and routine isolate analysis | High (user-friendly) | Limited error correction | [23–25] |
| **Velvet** | Legacy de Bruijn graph assembler; assembly quality is strongly dependent on k-mer choice, coverage, and parameter optimization | Variable; parameter-dependent | Configurable; supports paired-end and mate-pair data | Requires careful parameter optimization and can be memory-intensive | Historical short-read assembly method; not a current default recommendation for bacterial WGS-AMR workflows | Low to moderate (requires parameter tuning) | Basic error correction included | [26,27] |
| **ABySS**** | Scalable short-read assembler with distributed-computing support; contiguity and error profiles are dataset- and parameter-dependent | Variable; may require substantial computational setup | Distributed assembly tasks and selected bacterial datasets where such infrastructure is justified | Setup can be complex and memory demand can be high; less commonly used in routine bacterial AMR workflows | Large-scale assembly tasks and selected bacterial datasets requiring distributed computation | Moderate (requires expertise) | Advanced error correction | [28,29] |
| **SOAPdenovo** | Legacy short-read de Bruijn graph assembler; assembly quality is dataset- and parameter-dependent | Variable; may be efficient for selected short-read datasets | Historical short-read assembly method; not a current default recommendation for bacterial WGS-AMR workflows | May produce fragmented assemblies and is less suitable for extended-read or structurally complex bacterial datasets | Short-read assembly of datasets with low to moderate assembly complexity | Moderate | Partial error correction | [30–32] |

***** N50 summarizes assembly contiguity but does not confirm correctness. It should be interpreted alongside misassembly metrics (e.g., QUAST), microbial completeness and contamination assessment using CheckM/CheckM2, with BUSCO used only as an optional marker-gene completeness indicator, and read-mapping evidence, particularly when AMR interpretation depends on plasmid or MGE structure and junction accuracy.

****** ABySS has limited utility in hybrid assembly workflows for bacterial genomes.

**- Note:** Performance descriptors in this table are intended as task- and dataset-dependent summaries rather than universal rankings. Assembly output depends on read type, genome complexity, sequencing depth, library preparation, parameter settings, and validation method. For WGS-AMR interpretation, contiguity metrics should be interpreted together with misassembly assessment, read-backed validation, plasmid/MGE resolution, and downstream AMR-reporting requirements.

**Supplementary Table S3.** Long-read assembly tools and ONT-linked workflows relevant to AMR genomic-context interpretation.

| **Tool** | **Type of assembly / platform** | **Built-in error correction** | **Requires polishing** | **Key features** | **Main restrictions** | **Typical output**  **/ interpretation note** | **Ref.** |
| --- | --- | --- | --- | --- | --- | --- | --- |
| **Miniasm** | De novo assembly / ONT, PacBio | No | Yes | - Ultra-fast and lightweight assembler for rapid draft bacterial assemblies - Requires minimal computational resources | - Relies on external polishing and error correction - Lower consensus accuracy than corrected assemblers | Draft-level output; external correction and polishing are required before AMR-sensitive interpretation | [33,34] |
| **Flye** | De novo assembly / ONT, PacBio | Yes | Yes | - Robust long-read assembler for bacterial chromosomes and plasmids, including repeat-rich regions relevant to AMR context | - Additional polishing is often required, especially in repetitive or error-prone regions - Performance depends on read depth and assembly quality | Commonly used for bacterial long-read assembly; final reliability depends on read depth, chemistry, polishing, and validation | [35,36] |
| **PlasCAT** | Plasmid-focused assembly / long-read data | Yes | Yes | - Designed for plasmid reconstruction from long-read sequencing data - Distinguishes plasmid from chromosomal contigs and identifies AMR and virulence genes in plasmids | - Limited to plasmid-oriented analysis and not intended for complete microbial genome assembly - Computational demand may increase with large datasets | Plasmid-focused output; interpretation depends on long-read quality, plasmid complexity, and validation | [37] |
| **Canu** | De novo assembly / ONT, PacBio | Yes | Yes | - Strong correction of noisy long reads - Supports high-quality assembly of bacterial chromosomes and plasmids | - Computationally intensive, with high memory and CPU requirements - Slower on large datasets | Can support accurate long-read assembly when sufficient coverage and computational resources are available | [33,38] |
| **SDquest/**  **ABruijn** | De novo assembly / long-read data | Yes (integrated) | Yes | - Fast and resource-efficient for error-prone long reads - Suitable for bacterial datasets of moderate complexity | - Performance may be lower with small genomes or low-quality input reads - Assembly quality depends on read quality and coverage | Performance is dataset-dependent and should be validated against assembly metrics and read support | [32,39,40] |
| **Trycycler** | Consensus assembly from multiple long-read assemblies | No | Yes | - Combines multiple long-read assemblies, resolves discrepancies, and improves consensus accuracy - Supports circularization and refinement of bacterial genome assemblies | - Requires multiple near-complete input assemblies and high coverage - Manual checkpoints reduce suitability for high-throughput workflows - Less effective when repeats exceed read length | Consensus output can improve assembly confidence when multiple high-quality input assemblies and sufficient read depth are available | [41] |
| **Autocycler** | Automated consensus assembly from multiple long-read assemblies | No | Yes | - Automates consensus generation with minimal manual intervention - Includes diagnostic and visualization utilities and supports batch processing | - Works best with complete or near-complete input assemblies - Performance declines when repeats exceed read length | Automated consensus output; reliability depends on quality and completeness of input assemblies | [42] |
| **FALCON** | De novo assembly / PacBio | Yes (optional) | Yes | - Effective for PacBio long reads and integrated correction workflows - Supports high-quality assembly when PacBio data are available | - Computationally demanding - Primarily optimized for PacBio and less applicable to ONT workflows | PacBio-focused output; performance depends on read type, coverage, correction strategy, and computational resources | [43] |
| **Shasta** | De novo assembly / ONT | Yes (integrated) | No | - Fast long-read assembler designed for ONT data - Useful for rapid bacterial genome assembly | - Requires substantial memory - Supports long-read data only and does not provide hybrid assembly support | Fast ONT-focused assembly output; suitability for bacterial AMR interpretation depends on polishing and validation | [44] |
| **Redbean / wtdbg2** | De novo assembly/ONT | Partial | Yes | - Rapid draft assembly with relatively low computational demand - Useful for preliminary ONT-based bacterial analyses | - Lower consensus accuracy than more mature assemblers - Usually requires polishing before AMR-sensitive interpretation - Best suited to draft rather than final high-quality assemblies | Draft-oriented output; polishing and read-backed validation are required before AMR-sensitive interpretation | [45] |
| **Raven** | De novo assembly / long-read data | Yes | Yes (extra polishing required) | - Fast, simple, and resource-efficient - Suitable for small- to medium-sized bacterial genomes | - May exhibit reduced efficacy in genomes characterized by high repetitiveness or in datasets abundant with complex plasmids. - Less optimized for very large datasets | Fast long-read assembly output; final reliability depends on repeat structure, read quality, polishing, and validation | [46] |
| **EPI2ME** | ONT-linked analysis platform / cloud-based workflows | Yes (integrated) | Yes | - Supports real-time analysis, automated workflows, and AMR-related applications in ONT datasets - Useful for ONT-linked pathogen genome analysis workflows in clinical or surveillance settings - Integrates multiple tools within one platform | - Dependent on ONT-linked workflows and cloud infrastructure - Costs may increase in high-throughput settings | Not an assembler / workflow-dependent | [47–50] |

**- Notes:** This table summarizes long-read workflows that support AMR interpretation through plasmid reconstruction, MGE localization, repeat resolution, and structural context. These tools are not direct AMR gene- or mutation-detection tools; AMR-specific resources are summarized in **Table 2**. EPI2ME is included as an ONT-linked workflow/platform environment, not as a primary assembler, and its outputs should be reported with the selected workflow, software/database versions, ONT chemistry, basecaller model, and key parameters.

**Supplementary Table S4.** Hybrid assembly workflows relevant to AMR genomic-context interpretation.

| **Tool** | **Type of assembly** | **Input data** | **Key features** | **Requires polishing** | **Error correction** | **Key limitation** | **Ref.** |
| --- | --- | --- | --- | --- | --- | --- | --- |
| **SPAdes*** | Short-read assembly with optional hybrid mode | Illumina short reads, with optional long-read support | - Commonly used modular assembler for bacterial genomes - Supports bacterial short-read assembly, with optional hybrid functionality through hybridSPAdes - Effective for small bacterial genomes and variant-sensitive analysis | Usually no for short-read assemblies; hybrid outputs may still benefit from refinement | Yes (built-in short-read error correction) | - Computational demand increases with larger datasets - Memory use can be substantial | [31] |
| **MaSuRCA** | Hybrid assembly | Short reads (Illumina) + long reads (ONT, PacBio) | - Uses super-read strategies that can improve hybrid assembly contiguity in suitable datasets - Useful when long-read support is needed to improve bacterial genome reconstruction | Yes | Yes | - Long runtimes - Requires more complex configuration than bacteria-oriented hybrid tools | [51,52] |
| **Unicycler** | Hybrid assembly | Short reads (Illumina) + long reads (ONT, PacBio) | - Combines short- and long-read data to support bacterial genome assembly, including chromosome and plasmid reconstruction when input data quality is adequate - Can support chromosome and plasmid reconstruction in bacterial isolates when input data quality is adequate | Yes  (often minimal additional polishing) | Yes (integrated) | - Best suited to bacterial genomes rather than large non-bacterial assemblies - Requires short-read input for optimal performance | [51,53] |
| **OPERA-LG** | Hybrid scaffolding | Illumina + long reads (ONT, PacBio) | - Can improve scaffold contiguity using hybrid data, depending on the quality of the initial assembly and long-read support - Useful for extending bacterial assemblies when long-range structure is needed | No | No (scaffolding only) | - Primarily a scaffolding tool rather than a complete hybrid assembly workflow | [54,55] |
| **npScarf** | Hybrid scaffolding | Illumina + long reads (ONT) | - Lightweight hybrid scaffolding tool for improving selected draft assemblies - Useful for improving contiguity in bacterial genome assemblies | No | No (scaffolding only) | - Limited error correction and strongly dependent on the quality of the initial assembly | [56,57] |
| **PBcR** | De novo assembly using PacBio reads | PacBio long reads | - Supports PacBio long-read assembly with correction; current relevance depends on sequencing chemistry and workflow context - Useful for improving assembly continuity when PacBio reads are available | Yes | Yes, it corrects systematic errors in PacBio reads | - Computationally demanding and less commonly used in routine bacterial hybrid workflows | [34,58] |
| **Nanocorr** | Hybrid correction / post-assembly refinement | Error-prone assemblies with short-read support | - Can improve assembly accuracy through short-read-guided correction when correction reads and the initial assembly are suitable | Yes | Yes | - Performance depends strongly on the quality of the initial assembly and correction data | [56,59] |
| **NaS** | Hybrid assembly combining short and long reads | Short reads (Illumina) and long reads (PacBio or ONT) | - Combines short-and long-read information to support hybrid assembly; final output depends on input quality and data balance | Yes, polishing may be required to correct errors | Yes, supports error correction using both short- and long-read data | - Assembly quality depends strongly on the quality and balance of the input datasets | [56,60] |

* SPAdes is primarily a de Bruijn graph assembler for short-read data, with optional hybrid functionality through hybridSPAdes. It includes built-in error correction and specialized modes such as plasmidSPAdes and metaSPAdes.

**Note:** This table summarizes hybrid assembly, scaffolding, and correction workflows that may support AMR interpretation when structural context and base-level accuracy are required. These workflows are not direct AMR gene- or chromosomal mutation-detection tools; AMR-specific prediction resources are summarized separately in **Table 2** of the main manuscript.

**Supplementary Table S5.** Illustrative taxon-linked applicability of selected AMR resources across representative clinical and One Health taxa.

| **Representative taxon / setting** | **CARD** | **AMRFinderPlus** | **ResFinder** | **PlasmidFinder** | **PointFinder** | **NDARO** | **SARG** | **MEGARes** | **ARGminer** | **BacMet** | **DeepARG** | **BV-BRC (formerly PATRIC)** | **ARG-ANNOT** |
| --- | --- | --- | --- | --- | --- | --- | --- | --- | --- | --- | --- | --- | --- |
| ***Klebsiella pneumoniae*** | **+++** | **+++** | **+++** | **+++** | **+** | **+++** | **+** | **+** | **++** | **+** | **+** | **++** | **++** |
| ***Escherichia coli*** | **+++** | **+++** | **+++** | **+++** | **+++** | **+++** | **+** | **+** | **++** | **+** | **+** | **++** | **++** |
| ***Staphylococcus aureus*** | **+++** | **+++** | **+++** | **—** | **++** | **+++** | **+** | **+** | **++** | **+** | **+** | **++** | **++** |
| ***Acinetobacter baumannii*** | **+++** | **+++** | **+++** | **+** | **—** | **+++** | **+** | **+** | **++** | **+** | **+** | **++** | **++** |
| ***Pseudomonas aeruginosa*** | **+++** | **+++** | **+++** | **+** | **—** | **+++** | **+** | **+** | **++** | **+** | **+** | **++** | **++** |
| ***Salmonella enterica*** | **+++** | **+++** | **+++** | **+++** | **+++** | **+++** | **+** | **+** | **++** | **+** | **+** | **++** | **++** |
| ***Campylobacter jejuni*** | **++** | **++** | **++** | **—** | **+++** | **++** | **+** | **+** | **+** | **+** | **+** | **+** | **+** |
| ***Neisseria gonorrhoeae*** | **++** | **++** | **++** | **—** | **+** | **++** | **—** | **—** | **+** | **—** | **—** | **+** | **+** |
| ***Mycobacterium tuberculosis*** | **++** | **++** | **+** | **—** | **—** | **++** | **—** | **—** | **+** | **—** | **—** | **+** | **+** |
| **Environmental metagenomes / wastewater** | **+** | **+** | **+** | **—** | **—** | **+** | **+++** | **+++** | **++** | **+++** | **+++** | **+** | **—** |

**Note:** Legend: **+++** refers to strong/primary applicability under typical workflows; **++** refers to moderate applicability or commonly usable with some limitations; **+** refers to limited, indirect, or context-dependent applicability; — refers to not a primary resource or limited support for that organism or analytical function. This matrix is intended as an illustrative guide to typical applicability and scope, not as a measure of equivalent performance, taxon-specific validation, or clinical reliability.

**Supplementary Table S6:** Genome-analysis tools relevant to WGS-AMR workflows, including alignment, basecalling, polishing, variant calling, contamination screening, visualization, phylogenetic context, and mobile genetic element analysis.

| **Procedure** | **Tool** | **Primary purpose** | **Key features** | **Ref.** |
| --- | --- | --- | --- | --- |
| **Read Alignment** | **Minimap2** | Aligns short and long reads to reference genomes or databases | Fast and memory-efficient; supports short- and long-read alignment and multiple preset modes | [61] |
|  | **BWA-MEM** | Aligns short reads to reference genomes and is widely used in NGS workflows | Supports paired-end alignment, handles indels, and is widely used for Illumina data | [62] |
|  | **Bowtie2** | Aligns short reads and is designed for fast and memory-efficient alignment | Highly efficient for short reads, supports gapped alignment, and handles paired-end data | [63] |
|  | **LAST** | Sensitive alignment for long, error-prone reads, including Nanopore and PacBio data | Effective for mapping large genomes, sensitive to indels, and useful for challenging alignment contexts | [64] |
|  | **pbmm2** | PacBio-optimized alignment of HiFi, CLR, Iso-Seq, and related reads to reference genomes | Built on minimap2 with PacBio-specific presets; outputs BAM suitable for polishing, variant calling, transcript mapping, and SMRT Link workflows | [65,66] |
| **Base Calling** | **Guppy** | Converts raw ONT electrical signals into nucleotide sequences | Base calling supports GPU acceleration and integration with Nanopore sequencing pipelines | [67] |
|  | **Bonito** | Open-source ONT basecaller for nanopore signal-to-sequence conversion | Uses neural-network models for nanopore signal-to-sequence conversion; performance depends on chemistry, model version, and workflow context | [67] |
|  | **Albacore** | Legacy ONT base caller that converts raw signal into nucleotide sequence | Used in earlier Nanopore workflows and largely replaced by newer ONT basecalling tools | [67] |
|  | **Dorado** | Supports POD5/FAST5 input, FASTQ or BAM output, demultiplexing, adapter trimming, duplex workflows, and modified-base tagging; results should be reported with model and software version | Automatically selects appropriate models, supports POD5/FAST5 input, outputs FASTQ or BAM files, and includes options for methylation tagging, demultiplexing, and adapter trimming | [68] |
|  | **ONT R10.4.1 / Kit 14**^1^ | ONT chemistry and library-kit combination used in recent nanopore sequencing workflows | Can support improved read accuracy relative to earlier ONT generations when paired with appropriate Dorado models, adequate coverage, and validated workflows | [69,70] |
|  | **SMRTbell**^1^ | Circular DNA library construct used in PacBio SMRT sequencing to enable multiple passes over the same insert | Supports CCS/HiFi generation, barcoding, multiplexing, and high-accuracy PacBio sequencing workflows | [61,66,71–73] |
|  | **SMRT Link** | PacBio analysis suite for run setup, CCS/HiFi generation, mapping, variant calling, and downstream analysis | Provides GUI and CLI modules for alignment, variant calling, Iso-Seq, target enrichment, and methylation analysis | [66,74] |
| **Polishing** | **Pilon** | Corrects errors in genome assemblies, improving base-level accuracy | Uses short-read data to correct indels, local errors, and consensus sequence inaccuracies | [75] |
|  | **Racon** | Consensus sequence polishing using long reads | Efficient for fast, multiple-round polishing and integrates well with Minimap2 for assembly correction | [33] |
|  | **Medaka** | Deep-learning-based polishing tool for ONT reads | Provides ONT-focused polishing using neural-network models; performance depends on chemistry, model version, and workflow context | [67] |
|  | **NanoPolish** | Polishes assemblies using raw signal data from Nanopore sequencing | Aligns raw signals to improve consensus sequences and can detect methylation and other modifications | [36,76,77] |
|  | **POLCA** | Fast short-read-based correction of small assembly errors | Uses short-read alignments to correct small errors in assembled genomes | [78] |
|  | **NextPolish2** | Iterative polishing tool for short-read and long-read assemblies, including repeat-aware refinement of HiFi-based assemblies | Uses alignment and *k*-mer-guided correction to improve SNV and indel accuracy while limiting overcorrection | [79,80] |
|  | **Homopolish** | Corrects ONT homopolymer and related systematic errors using homologous sequences | Helps reduce systematic errors, including homopolymer-associated errors, in selected nanopore assemblies | [81] |
|  | **MarginPolish/HELEN** | Polishes ONT draft assemblies to reduce homopolymer and small indel errors after long-read assembly | Combines graph-based consensus refinement and neural prediction to improve nanopore-only assembly accuracy | [44] |
|  | **Polypolish** | Uses Illumina short reads to polish long-read bacterial assemblies, especially for small indels and homopolymer errors | Uses all-per-read alignments to improve polishing in repeat regions and is often combined with tools such as POLCA | [82] |
| **Annotation** | **Prokka** | Rapid annotation of prokaryotic genomes, identifying genes, CDS, RNAs, and more | Automates the annotation process and supports multiple databases for gene prediction | [83] |
|  | **RAST** | Automated service for annotation of bacterial and archaeal genomes | Provides functional annotation and pathway-oriented interpretation for bacterial and archaeal genomes | [84] |
| **Variant**  **Calling** | **GATK** | Comprehensive toolkit for detecting variants, including SNPs, indels, and selected structural variants | Provides a widely used framework for variant discovery, filtering, and refinement in NGS workflows | [85] |
|  | **FreeBayes** | Bayesian variant caller for SNPs, indels, and complex variants | Supports complex variants, including indels, and is useful for population-level variant calling | [86] |
|  | **DeepVariant** | Uses deep learning to call variants from short- or long-read sequence data, depending on model and workflow | Neural network-based variant calling for short- and long-read data; performance depends on sequencing platform, model selection, training data, and validation context | [87] |
| **Phylogenetic Analysis** | **MEGA** | Software for sequence analysis, phylogenetic reconstruction, and evolutionary interpretation | Supports alignment, model selection, and phylogenetic tree construction in a user-friendly environment | [88] |
|  | **IQ-TREE** | Maximum-likelihood phylogenetic inference software suitable for large genomic datasets | Handles large datasets, supports multiple sequence-evolution models, and is suitable for bootstrap analysis | [89] |
|  | **RAxML** | Effective techniques for constructing phylogenetic trees from extensive datasets | Supports maximum-likelihood phylogenetic inference for large datasets, with runtime and memory usage depending on dataset size and model parameters. | [90] |
|  | **FastANI** | Fast genome-to-genome average nucleotide identity (ANI) computation for clustering and dereplication | Alignment-free ANI estimation designed for comparing closely related genomes and handling large datasets efficiently. | [91] |
| **Mobile Genetic Element Analysis** | **geNomad** | Identifies and annotates viruses and plasmids; detects proviruses integrated in host genomes | Useful for prophage-rich isolate assemblies and metagenomes; complements bacterial mobilome annotation alongside plasmid/ICE/IS resources | [92] |

^1^ ONT R10.4.1 / Kit 14 and SMRTbell are included because sequencing chemistry, kit configuration, and library design can materially influence base-calling accuracy, consensus quality, and downstream genomic interpretation; however, they should be understood as workflow components rather than standalone analytical tools.

- Note: This table summarizes broader genome-analysis support tools relevant to WGS-AMR workflows. These tools are not direct AMR prediction resources. AMR gene detection, chromosomal mutation inference, and AMR database-supported prediction tools are summarized separately in Table 2 of the main manuscript.

**Supplementary Table S7:** Practical scope and surveillance use of selected AMR resources across human, animal, and environmental sectors.

| **Resource** | **Sectors*** | **Typical surveillance outputs** | **Main limitation / note** |
| --- | --- | --- | --- |
| **ResFinder** | **H, A** | Presence and identity of acquired ARGs per isolate or cluster | Limited mutation support unless paired with PointFinder |
| **CARD** | **H, A** | ARO-linked AMR gene and mechanism annotations; model-informed resistance interpretation | Ontology mapping may still be needed for cross-database harmonization |
| **PlasmidFinder** | **H, A, E** | Plasmid replicon types (e.g., IncF, IncI) per isolate or cluster | Replicon typing alone does not confirm plasmid localization of ARGs |
| **AMRFinderPlus (NCBI)** | **H, A** | Gene/allele calls (e.g., *bla*CTX-M) and selected mutation calls (e.g., *gyrA* S83L) | Performance depends on assembly quality, curation depth, and database version |
| **PointFinder** | **H, A** | Curated resistance-associated point mutations (e.g., in *gyrA*, *parC*, *rpoB*) | Organism- and drug-specific mutation panels vary by species |
| **NDARO (NCBI)** | **H, A** | Linked AMR genotypes, selected MIC/AST associations, and related metadata | Availability and depth of phenotype-linked information vary across species |
| **SARG** | **E** | ARG abundance indices and environmental resistome profiles | Primarily aligned with environmental surveillance; clinical interpretation is indirect |
| **MEGARes** | **A, E** | Counts of AMR classes, mechanisms, and genes from metagenomic datasets | Best suited to metagenomic workflows; limited support for isolate-level chromosomal SNP interpretation |
| **BacMet** | **A, E** | Markers of metal and biocide resistance relevant to co-selection analyses | Not an antibiotic ARG database; intended to complement AMR profiling rather than replace it |
| **DeepARG** | **H, A, E** | Probabilistic ARG assignments from genomic or metagenomic data | Outputs are model-dependent and influenced by model version and training data |
| **BV-BRC (formerly PATRIC)** | **H, A** | Typing, phylogenomics, and integrated AMR-related genome analysis | Broad pathogen genomics platform; not a specialized AMR reference database |
| **ARG-ANNOT** | **H** | ARG presence and identity for established resistance determinants | Legacy resource with limited update continuity; best used as a supplementary reference |

**Note:** This table complements Table 2 in the main manuscript by summarizing typical surveillance applications of selected AMR resources across human, animal, and environmental sectors. It is intended to clarify practical use contexts rather than compare equivalent performance across resources. References for the resources summarized here correspond to those provided in Table 2 of the main manuscript.
*** H = human; A = animal; E = environmental.**

**Supplementary Table S8:** Molecular identification markers and their relevance to AMR/WGS workflows.

| **Marker** | **Possible Assay** | **Primary use in AMR/WGS workflows** | **Key limitations** | **Ref.** |
| --- | --- | --- | --- | --- |
| ***IS6110***  ***(M. tuberculosis)*** | PCR/qPCR detection of the Mycobacterium tuberculosis complex-associated insertion sequence IS6110 | Confirms *M. tuberculosis* prior to WGS/AMR profiling; anchors database metadata | Copy number varies, and rare low-copy strains may decrease sensitivity | [93] |
| ***wzi*/K-locus ^1^ *(Klebsiella)*** | Capsule gene (*wzi*) and complete K-locus typing | Capsule (KL) assignment connects virulence and epidemiology with the AMR context | *wzi* typing may be less accurate than full K-locus assignment, and recombination can affect KL calls | [94,95] |
| **MLST**  ***(Multi-locus sequence typing)*** | Sequence type (ST) of seven housekeeping loci | Portable epidemiology, tracking clonal lineages, and enabling cross-study comparability | Not adequate for detailed outbreak resolution compared to cgMLST/*WGS* SNPs | [96] |
| ***spa* typing ^2^** | Repeating polymorphs in the *spa* X-region  (*S. aureus*) | Rapid single-locus typing method for *S. aureus* surveillance | Single-locus homoplasy or recombination can obscure phylogenetic relationships | [97] |
| ***gyrB*** | DNA gyrase subunit B gene | Species delineation and phylogeny when 16S results are ambiguous | Varies among different taxa; recombination occurs occasionally | [98,99] |
| **16S rRNA** | Conserved gene containing variable regions (V1–V9) | Provides broad bacterial identification and preliminary taxonomic classification for isolates and metagenomes | Some complexes, such as the *K. pneumoniae* complex, have limited resolution at the species or strain level | [100] |
| **ITS (16S–23S)** | Ribosomal intergenic spacer | Finer taxonomic resolution than 16S in many genera | Multiple operons and intragenomic variants complicate analysis | [101,102] |
| ***rpoB* ^3^** | RNA polymerase β-subunit gene | Species identification and, in M. tuberculosis, screening of rifampicin resistance-associated hotspots | Species-identification performance is taxon-dependent, and resistance inference requires organism-specific interpretation and confirmatory testing where clinically relevant | [103,104] |

**^1^** K-locus assignment is frequently conducted using Kaptive; KL types are utilized for epidemiological studies.
**^2^** *Spa* typing is a method specifically used for identifying strains of *S. aureus.*
**^3^** RRDR refers to the rifampicin resistance–determining region in *rpoB*.

**Supplementary Figure S1**

**Supplementary Figure S1.** Workflow from molecular identification to database-supported AMR surveillance analysis**.**
The schematic shows a four-stage pipeline: (1) Molecular identification with species/lineage markers to confirm the pathogen and gather typing data; (2) Genomic resistance profiling with curated resources to detect resistance genes, alleles, and mutations; (3) Incorporation into AMR repositories with metadata (host, source, time, location); and (4) Surveillance and analytics. Arrows show data flow; the framework is platform-agnostic, compatible with short- and long-read sequencing, and supports hybrid assemblies. Abbreviations: MLST, multi-locus sequence typing; ITS, internal transcribed spacer.

**Supplementary Table S9:** Representative web resources for the AMR databases and platforms discussed in the manuscript.

| **Resource/Database** | **Link** |
| --- | --- |
| **ARGminer** | https://github.com/gaarangoa/ARGminer &  https://bio.tools/argminer |
| **BacMet** | http://bacmet.biomedicine.gu.se &  https://ngdc.cncb.ac.cn/databasecommons/database/id/254 |
| **CARD (Comprehensive Antibiotic Resistance Database)** | https://card.mcmaster.ca |
| **AMRFinderPlus** | https://www.ncbi.nlm.nih.gov/pathogens/antimicrobial-resistance/AMRFinder |
| **ResFinder** | https://cge.cbs.dtu.dk/services/ResFinder &  https://cge.food.dtu.dk/services/ResFinder/ |
| **PlasmidFinder** | https://github.com/genomicepidemiology/plasmidfinder and  https://cge.food.dtu.dk/services/PlasmidFinder |
| **NDARO (National Database of Antibiotic-Resistant Organisms)** | https://www.ncbi.nlm.nih.gov/pathogens/antimicrobial-resistance |
| **SARG (Structured ARGs)** | https://smile.hku.hk/ARGs/Indexing/download &  https://github.com/xinehc/args_oap |
| **MEGARes** | https://www.meglab.org &  https://github.com/meglab-metagenomics/amrplusplus_v2 |
| **DeepARG** | https://bio.tools/deeparg &  https://github.com/gaarangoa/deeparg |
| **PointFinder** | https://bitbucket.org/genomicepidemiology/pointfinder_db.git/src &  https://bitbucket.org/genomicepidemiology/pointfinder |
| **BV-BRC (formerly PATRIC)** | https://www.bv-brc.org & https://www.patricbrc.org |

**Note:** Links were selected to prioritize official database, service, repository, or platform pages. Publication links are provided in the reference list and are not repeated here unless they represent the most stable access route. BV-BRC is listed as the active platform, while PATRIC is retained only as a legacy/former resource. DeepARG and ARGs-OAP are listed with repository links to improve reproducibility.

**References for Supplementary:**

[1]	Andrews S, Krueger F, Segonds-Pichon A, Biggins L, Krueger C, Wingett S. FastQC: A quality control tool for high throughput sequence data 2010. https://www.bioinformatics.babraham.ac.uk/projects/fastqc/ (accessed May 12, 2026).

[2]	Patel RK, Jain M. NGS QC toolkit: A toolkit for quality control of next generation sequencing data. PLoS One 2012;7:e30619–e30619. https://doi.org/10.1371/journal.pone.0030619.

[3]	Chen S, Huang T, Zhou Y, Han Y, Xu M, Gu J. AfterQC: Automatic filtering, trimming, error removing and quality control for fastq data. BMC Bioinformatics 2017;18:80. https://doi.org/10.1186/s12859-017-1469-3.

[4]	Chen S. Ultrafast one-pass FASTQ data preprocessing, quality control, and deduplication using fastp. IMeta 2023;2:e107. https://doi.org/10.1002/imt2.107.

[5]	Chen S, Zhou Y, Chen Y, Gu J. Fastp: An ultra-fast all-in-one FASTQ preprocessor. Bioinformatics 2018;34:i884–90. https://doi.org/10.1093/bioinformatics/bty560.

[6]	Martin M. Cutadapt removes adapter sequences from high-throughput sequencing reads. EMBnetJournal 2011;17:10. https://doi.org/10.14806/ej.17.1.200.

[7]	Bolger AM, Lohse M, Usadel B. Trimmomatic: A flexible trimmer for Illumina sequence data. Bioinformatics 2014;30:2114–20. https://doi.org/10.1093/bioinformatics/btu170.

[8]	De Coster W, D’Hert S, Schultz DT, Cruts M, Van Broeckhoven C. NanoPack: visualizing and processing long-read sequencing data. Bioinformatics 2018;34:2666–9. https://doi.org/10.1093/bioinformatics/bty149.

[9]	Fukasawa Y, Ermini L, Wang H, Carty K, Cheung MS. LongQC: A quality control tool for third generation sequencing long read data. G3 Genes, Genomes, Genet 2020;10:1193–6. https://doi.org/10.1534/g3.119.400864.

[10]	Bonenfant Q, Noe L, Touzet H. Porechop ABI: Discovering unknown adapters in Oxford Nanopore Technology sequencing reads for downstream trimming. Bioinforma Adv 2023;3:vbac085. https://doi.org/10.1093/bioadv/vbac085.

[11]	Wick R, Volkening J. Porechop: adapter trimmer for Oxford Nanopore reads. GitHub repository. Available from: https://github.com/rrwick/Porechop. Accessed 12 May 2026.

[12]	De Coster W, Rademakers R. NanoPack2: population-scale evaluation of long-read sequencing data. Bioinformatics 2023;39:btad311. https://doi.org/10.1093/bioinformatics/btad311.

[13]	Chen Y, Chen Y, Shi C, Huang Z, Zhang Y, Li S, et al. SOAPnuke: A MapReduce acceleration-supported software for integrated quality control and preprocessing of high-throughput sequencing data. Gigascience 2018;7:1–6. https://doi.org/10.1093/gigascience/gix120.

[14]	Yan L, Yin Z, Zhang H, Zhao Z, Wang M, Müller A, et al. RabbitQCPlus 2.0: More efficient and versatile quality control for sequencing data. Methods 2023;216:39–50. https://doi.org/10.1016/j.ymeth.2023.06.007.

[15]	Low AJ, Koziol AG, Manninger PA, Blais B, Carrillo CD. ConFindr: rapid detection of intraspecies and cross-species contamination in bacterial whole-genome sequence data. PeerJ 2019;7:e6995. https://doi.org/10.7717/peerj.6995.

[16]	Wood DE, Lu J, Langmead B. Improved metagenomic analysis with Kraken 2. Genome Biol 2019;20:257. https://doi.org/10.1186/s13059-019-1891-0.

[17]	Seppey M, Manni M, Zdobnov EM. BUSCO: Assessing genome assembly and annotation completeness. Methods Mol Biol 2019;1962:227–45. https://doi.org/10.1007/978-1-4939-9173-0_14.

[18]	Waterhouse RM, Seppey M, Simão FA, Manni M, Ioannidis P, Klioutchnikov G, et al. BUSCO Applications from Quality Assessments to Gene Prediction and Phylogenomics. Mol Biol Evol 2018;35:543–8. https://doi.org/10.1093/molbev/msx319.

[19]	Parks DH, Imelfort M, Skennerton CT, Hugenholtz P, Tyson GW. CheckM: assessing the quality of microbial genomes recovered from isolates, single cells, and metagenomes. Genome Res 2015;25:1043–55. https://doi.org/10.1101/gr.186072.114.

[20]	Chklovski A, Parks DH, Woodcroft BJ, Tyson GW. CheckM2: a rapid, scalable and accurate tool for assessing microbial genome quality using machine learning. Nat Methods 2023;20:1203–12. https://doi.org/10.1038/s41592-023-01940-w.

[21]	Mikheenko A, Saveliev V, Hirsch P, Gurevich A. WebQUAST: Online evaluation of genome assemblies. Nucleic Acids Res 2023;51:W601–6. https://doi.org/10.1093/nar/gkad406.

[22]	Seemann T. Shovill: faster SPAdes assembly of Illumina reads. GitHub repository. Available from: https://github.com/tseemann/shovill. Accessed 12 May 2026.

[23]	Mariano DCBB, Pereira FL, Aguiar EL, Oliveira LC, Benevides L, Guimarães LC, et al. SIMBA: A web tool for managing bacterial genome assembly generated by Ion PGM sequencing technology. BMC Bioinformatics 2016;17:456. https://doi.org/10.1186/s12859-016-1344-7.

[24]	Agarwal T, Suravajhala R, Bhushan M, Goswami P, Iddamalgoda L, Malik B, et al. Recent advances in gene and genome assembly: Challenges and implications. Adv Synth Biol, Singapore: Springer Singapore; 2020, p. 199–220. https://doi.org/10.1007/978-981-15-0081-7_12.

[25]	Souvorov A, Agarwala R, Lipman DJ. SKESA: Strategic k-mer extension for scrupulous assemblies. Genome Biol 2018;19:153. https://doi.org/10.1186/s13059-018-1540-z.

[26]	Zerbino DR, Birney E. Velvet: algorithms for de novo short read assembly using de Bruijn graphs. Genome Res 2008;18:821–9. https://doi.org/10.1101/gr.074492.107.

[27]	Zerbino DR. Using the Velvet de novo assembler for short-read sequencing technologies. Curr Protoc Bioinforma 2010;Chapter 11:Unit 11.5-Unit 11.5. https://doi.org/10.1002/0471250953.bi1105s31.

[28]	Jackman SD, Vandervalk BP, Mohamadi H, Chu J, Yeo S, Hammond SA, et al. ABySS 2.0: resource-efficient assembly of large genomes using a Bloom filter. Genome Res 2017;27:768–77. https://doi.org/10.1101/gr.214346.116.

[29]	Simpson JT, Wong K, Jackman SD, Schein JE, Jones SJM, Birol I. ABySS: a parallel assembler for short read sequence data. Genome Res 2009;19:1117–23. https://doi.org/10.1101/gr.089532.108.

[30]	Luo R, Liu B, Xie Y, Li Z, Huang W, Yuan J, et al. SOAPdenovo2: an empirically improved memory-efficient short-read de novo assembler. Gigascience 2012;1:2047-217X. https://doi.org/10.1186/2047-217X-1-18.

[31]	Bankevich A, Nurk S, Antipov D, Gurevich AA, Dvorkin M, Kulikov AS, et al. SPAdes: A new genome assembly algorithm and its applications to single-cell sequencing. J Comput Biol 2012;19:455–77. https://doi.org/10.1089/cmb.2012.0021.

[32]	Liu Y, Schmidt B, Maskell DL. Parallelized short read assembly of large genomes using de Bruijn graphs. BMC Bioinformatics 2011;12:354. https://doi.org/10.1186/1471-2105-12-354.

[33]	Vaser R, Sović I, Nagarajan N, Šikić M. Fast and accurate de novo genome assembly from long uncorrected reads. Genome Res 2017;27:737–46. https://doi.org/10.1101/gr.214270.116.

[34]	Berlin K, Koren S, Chin CS, Drake JP, Landolin JM, Phillippy AM. Assembling large genomes with single-molecule sequencing and locality-sensitive hashing. Nat Biotechnol. 2015;33:623–30. https://doi.org/10.1038/nbt.3238.

[35]	Liu H, Wu S, Li A, Ruan J. SMARTdenovo: a de novo assembler using long noisy reads. Gigabyte 2021;2021:1–9. https://doi.org/10.46471/gigabyte.15.

[36]	Kolmogorov M, Yuan J, Lin Y, Pevzner PA. Assembly of long, error-prone reads using repeat graphs. Nat Biotechnol 2019;37:540–6. https://doi.org/10.1038/s41587-019-0072-8.

[37]	Peccoud S, Berezin CT, Hernandez SI, Peccoud J. PlasCAT: Plasmid Cloud Assembly Tool. Bioinformatics 2024;40:btae299. https://doi.org/10.1093/bioinformatics/btae299.

[38]	Koren S, Walenz BP, Berlin K, Miller JR, Bergman NH, Phillippy AM. Canu: scalable and accurate long-read assembly via adaptive k -mer weighting and repeat separation. Genome Res 2017;27:722–36. https://doi.org/10.1101/gr.215087.116.

[39]	Georganas E, Buluç A, Chapman J, Oliker L, Rokhsar D, Yelick K. Parallel de Bruijn Graph Construction and Traversal for de Novo Genome Assembly. Int Conf High Perform Comput Networking, Storage Anal SC, vol. 2015- Janua, IEEE; 2014, p. 437–48. https://doi.org/10.1109/SC.2014.41.

[40]	Lin Y, Yuan J, Kolmogorov M, Shen MW, Chaisson M, Pevzner PA. Assembly of long error-prone reads using de Bruijn graphs. Proc Natl Acad Sci U S A 2016;113:E8396–405. https://doi.org/10.1073/pnas.1604560113.

[41]	Wick RR, Judd LM, Cerdeira LT, Hawkey J, Méric G, Vezina B, et al. Trycycler: consensus long-read assemblies for bacterial genomes. Genome Biol 2021;22:266. https://doi.org/10.1186/s13059-021-02483-z.

[42]	Wick RR, Howden BP, Stinear TP. Autocycler: long-read consensus assembly for bacterial genomes. Bioinformatics 2025;41:btaf474. https://doi.org/10.1093/bioinformatics/btaf474.

[43]	Chin C-S, Peluso P, Sedlazeck FJ, Nattestad M, Concepcion GT, Clum A, et al. Phased diploid genome assembly with single-molecule real-time sequencing. Nat Methods 2016;13:1050–4. https://doi.org/10.1038/nmeth.4035.

[44]	Shafin K, Pesout T, Lorig-Roach R, Haukness M, Olsen HE, Bosworth C, et al. Nanopore sequencing and the Shasta toolkit enable efficient de novo assembly of eleven human genomes. Nat Biotechnol 2020;38:1044–53. https://doi.org/10.1038/s41587-020-0503-6.

[45]	Ruan J, Li H. Fast and accurate long-read assembly with wtdbg2. Nat Methods 2020;17:155–8. https://doi.org/10.1038/s41592-019-0669-3.

[46]	Vaser R, Šikić M. Time- and memory-efficient genome assembly with RAVEN. Nat Comput Sci 2021;1:332–6. https://doi.org/10.1038/s43588-021-00073-4.

[47]	Oxford Nanopore Technologies. EPI2ME platform documentation 2026. https://epi2me.nanoporetech.com/ (accessed May 12, 2026).

[48]	Tyler AD, Mataseje L, Urfano CJ, Schmidt L, Antonation KS, Mulvey MR, et al. Evaluation of Oxford Nanopore’s MinION Sequencing Device for Microbial Whole Genome Sequencing Applications. Sci Rep 2018;8:10931. https://doi.org/10.1038/s41598-018-29334-5.

[49]	Kerkhof LJ. Is Oxford Nanopore sequencing ready for analyzing complex microbiomes? FEMS Microbiol Ecol 2021;97. https://doi.org/10.1093/femsec/fiab001.

[50]	Sung YH, Ju YK, Lee HJ, Park SM, Suh JW, Kim JY, et al. Clinical performance of real-time nanopore metagenomic sequencing for rapid identification of bacterial pathogens in cerebrospinal fluid: a pilot study. Sci Rep 2025;15:3493. https://doi.org/10.1038/s41598-025-87858-z.

[51]	Chen Z, Erickson DL, Meng J. Benchmarking long-read assemblers for genomic analyses of bacterial pathogens using oxford nanopore sequencing. Int J Mol Sci 2020;21:1–27. https://doi.org/10.3390/ijms21239161.

[52]	Zimin A V., Puiu D, Luo M-C, Zhu T, Koren S, Marçais G, et al. Hybrid assembly of the large and highly repetitive genome of Aegilops tauschii, a progenitor of bread wheat, with the MaSuRCA mega-reads algorithm. Genome Res 2017;27:787–92. https://doi.org/10.1101/gr.213405.116.

[53]	Wick RR, Judd LM, Gorrie CL, Holt KE. Unicycler: Resolving bacterial genome assemblies from short and long sequencing reads. PLoS Comput Biol 2017;13:e1005595. https://doi.org/10.1371/journal.pcbi.1005595.

[54]	Gao S, Bertrand D, Chia BKH, Nagarajan N. OPERA-LG: efficient and exact scaffolding of large, repeat-rich eukaryotic genomes with performance guarantees. Genome Biol 2016;17:102. https://doi.org/10.1186/s13059-016-0951-y.

[55]	Bertrand D, Shaw J, Kalathiyappan M, Ng AHQ, Kumar MS, Li C, et al. Hybrid metagenomic assembly enables high-resolution analysis of resistance determinants and mobile elements in human microbiomes. Nat Biotechnol 2019;37:937–44. https://doi.org/10.1038/s41587-019-0191-2.

[56]	Cao MD, Nguyen SH, Ganesamoorthy D, Elliott AG, Cooper MA, Coin LJM. Scaffolding and completing genome assemblies in real-time with nanopore sequencing. Nat Commun 2017;8:14515. https://doi.org/10.1038/ncomms14515.

[57]	Nguyen SH, Cao MD, Coin LJM. Real-time resolution of short-read assembly graph using ONT long reads. PLOS Comput Biol 2021;17:e1008586. https://doi.org/10.1371/journal.pcbi.1008586.

[58]	Koren S, Schatz MC, Walenz BP, Martin J, Howard JT, Ganapathy G, et al. Hybrid error correction and de novo assembly of single-molecule sequencing reads. Nat Biotechnol 2012;30:693–700. https://doi.org/10.1038/nbt.2280.

[59]	Goodwin S, Gurtowski J, Ethe-Sayers S, Deshpande P, Schatz MC, McCombie WR. Oxford Nanopore sequencing, hybrid error correction, and de novo assembly of a eukaryotic genome. Genome Res 2015;25:1750–6. https://doi.org/10.1101/gr.191395.115.

[60]	Madoui M-A, Engelen S, Cruaud C, Belser C, Bertrand L, Alberti A, et al. Genome assembly using Nanopore-guided long and error-free DNA reads. BMC Genomics 2015;16:327. https://doi.org/10.1186/s12864-015-1519-z.

[61]	Li H. Minimap2: Pairwise alignment for nucleotide sequences. Bioinformatics 2018;34:3094–100. https://doi.org/10.1093/bioinformatics/bty191.

[62]	Li H, Durbin R. Fast and accurate short read alignment with Burrows-Wheeler transform. Bioinformatics 2009;25:1754–60. https://doi.org/10.1093/bioinformatics/btp324.

[63]	Langmead B, Salzberg SL. Fast gapped-read alignment with Bowtie 2. Nat Methods 2012;9:357–9. https://doi.org/10.1038/nmeth.1923.

[64]	Kiełbasa SM, Wan R, Sato K, Horton P, Frith MC. Adaptive seeds tame genomic sequence comparison. Genome Res 2011;21:487–93. https://doi.org/10.1101/gr.113985.110.

[65]	Pacific Biosciences. pbmm2: a minimap2 frontend for PacBio native data formats. Version 1.17.0. GitHub repository. Available from: https://github.com/PacificBiosciences/pbmm2. Accessed 12 May 2026.

[66]	Biosciences P. SMRT Link User Guide v25.1. Version 25. Menlo Park, CA: Pacific Biosciences of California, Inc.; 2025.

[67]	Resources: Documentation | Oxford Nanopore Technologies n.d.

[68]	Oxford Nanopore Technologies. Dorado: Oxford Nanopore basecaller documentation. Available from: https://github.com/nanoporetech/dorado. Accessed 12 May 2026.

[69]	Sanderson ND, Hopkins KM V, Colpus M, Parker M, Lipworth S, Crook D, et al. Evaluation of the accuracy of bacterial genome reconstruction with Oxford Nanopore R10.4.1 long-read-only sequencing. Microb Genomics 2024;10. https://doi.org/10.1099/mgen.0.001246.

[70]	Dabernig-Heinz J, Lohde M, Hölzer M, Cabal A, Conzemius R, Brandt C, et al. A multicenter study on accuracy and reproducibility of nanopore sequencing-based genotyping of bacterial pathogens. J Clin Microbiol 2024;62:e0062824. https://doi.org/10.1128/jcm.00628-24.

[71]	Cuber P, Chooneea D, Geeves C, Salatino S, Creedy TJ, Griffin C, et al. Comparing the accuracy and efficiency of third generation sequencing technologies, Oxford Nanopore Technologies, and Pacific Biosciences, for DNA barcode sequencing applications. Ecol Genet Genomics 2023;28:100181. https://doi.org/10.1016/j.egg.2023.100181.

[72]	Wenger AM, Peluso P, Rowell WJ, Chang P-C, Hall RJ, Concepcion GT, et al. Accurate circular consensus long-read sequencing improves variant detection and assembly of a human genome. Nat Biotechnol 2019;37:1155–62. https://doi.org/10.1038/s41587-019-0217-9.

[73]	Travers KJ, Chin C-S, Rank DR, Eid JS, Turner SW. A flexible and efficient template format for circular consensus sequencing and SNP detection. Nucleic Acids Res 2010;38:e159. https://doi.org/10.1093/nar/gkq543.

[74]	Link S, Notes R. SMRT ® Link Release Notes (v10.1). Version 25. Pacific Biosciences of California, Inc.; 2021.

[75]	Walker BJ, Abeel T, Shea T, Priest M, Abouelliel A, Sakthikumar S, et al. Pilon: An integrated tool for comprehensive microbial variant detection and genome assembly improvement. PLoS One 2014;9:e112963. https://doi.org/10.1371/journal.pone.0112963.

[76]	Senol Cali D, Kim JS, Ghose S, Alkan C, Mutlu O. Nanopore sequencing technology and tools for genome assembly: computational analysis of the current state, bottlenecks and future directions. Brief Bioinform 2019;20:1542–59. https://doi.org/10.1093/bib/bby017.

[77]	Wick RR, Judd LM, Gorrie CL, Holt KE. Completing bacterial genome assemblies with multiplex MinION sequencing. Microb Genomics 2017;3:e000132. https://doi.org/10.1099/mgen.0.000132.

[78]	Zimin A V., Salzberg SL. The genome polishing tool POLCA makes fast and accurate corrections in genome assemblies. PLoS Comput Biol 2020;16:e1007981. https://doi.org/10.1371/journal.pcbi.1007981.

[79]	Hu J, Fan J, Sun Z, Liu S. NextPolish: a fast and efficient genome polishing tool for long-read assembly. Bioinformatics 2020;36:2253–5. https://doi.org/10.1093/bioinformatics/btz891.

[80]	Hu J, Wang Z, Liang F, Liu SL, Ye K, Wang DP. NextPolish2: A Repeat-aware Polishing Tool for Genomes Assembled Using HiFi Long Reads. Genomics, Proteomics Bioinforma 2024;22:qzad009. https://doi.org/10.1093/gpbjnl/qzad009.

[81]	Huang YT, Liu PY, Shih PW. Homopolish: a method for the removal of systematic errors in nanopore sequencing by homologous polishing. Genome Biol 2021;22:95. https://doi.org/10.1186/s13059-021-02282-6.

[82]	Wick RR, Holt KE. Polypolish: Short-read polishing of long-read bacterial genome assemblies. PLoS Comput Biol 2022;18:e1009802. https://doi.org/10.1371/journal.pcbi.1009802.

[83]	Seemann T. Prokka: Rapid prokaryotic genome annotation. Bioinformatics 2014;30:2068–9. https://doi.org/10.1093/bioinformatics/btu153.

[84]	Aziz RK, Bartels D, Best A, DeJongh M, Disz T, Edwards RA, et al. The RAST Server: Rapid annotations using subsystems technology. BMC Genomics 2008;9:75. https://doi.org/10.1186/1471-2164-9-75.

[85]	McKenna A, Hanna M, Banks E, Sivachenko A, Cibulskis K, Kernytsky A, et al. The genome analysis toolkit: A MapReduce framework for analyzing next-generation DNA sequencing data. Genome Res 2010;20:1297–303. https://doi.org/10.1101/gr.107524.110.

[86]	Garrison E, Marth G. Haplotype-based variant detection from short-read sequencing. arXiv preprint arXiv. 2012;1207.3907.

[87]	Poplin R, Chang PC, Alexander D, Schwartz S, Colthurst T, Ku A, et al. A universal snp and small-indel variant caller using deep neural networks. Nat Biotechnol 2018;36:983. https://doi.org/10.1038/nbt.4235.

[88]	Kumar S, Stecher G, Li M, Knyaz C, Tamura K. MEGA X: Molecular evolutionary genetics analysis across computing platforms. Mol Biol Evol 2018;35:1547–9. https://doi.org/10.1093/molbev/msy096.

[89]	Nguyen LT, Schmidt HA, Von Haeseler A, Minh BQ. IQ-TREE: A fast and effective stochastic algorithm for estimating maximum-likelihood phylogenies. Mol Biol Evol 2015;32:268–74. https://doi.org/10.1093/molbev/msu300.

[90]	Stamatakis A. RAxML version 8: A tool for phylogenetic analysis and post-analysis of large phylogenies. Bioinformatics 2014;30:1312–3. https://doi.org/10.1093/bioinformatics/btu033.

[91]	Jain C, Rodriguez-R LM, Phillippy AM, Konstantinidis KT, Aluru S. High throughput ANI analysis of 90K prokaryotic genomes reveals clear species boundaries. Nat Commun 2018;9:5114. https://doi.org/10.1038/s41467-018-07641-9.

[92]	Camargo AP, Roux S, Schulz F, Babinski M, Xu Y, Hu B, et al. Identification of mobile genetic elements with geNomad. Nat Biotechnol 2024;42:1303–12. https://doi.org/10.1038/s41587-023-01953-y.

[93]	Coros A, DeConno E, Derbyshire KM. IS6110, a Mycobacterium tuberculosis complex-specific insertion sequence, is also present in the genome of Mycobacterium smegmatis, suggestive of lateral gene transfer among mycobacterial species. J Bacteriol 2008;190:3408–10. https://doi.org/10.1128/JB.00009-08.

[94]	Brisse S, Passet V, Haugaard AB, Babosan A, Kassis-Chikhani N, Struve C, et al. Wzi gene sequencing, a rapid method for determination of capsulartype for klebsiella strains. J Clin Microbiol 2013;51:4073–8. https://doi.org/10.1128/JCM.01924-13.

[95]	Lam MMC, Wick RR, Judd LM, Holt KE, Wyres KL. Kaptive 2.0: updated capsule and lipopolysaccharide locus typing for the Klebsiella pneumoniae species complex. Microb Genomics 2022;8:800. https://doi.org/10.1099/mgen.0.000800.

[96]	Maiden MCJ, Bygraves JA, Feil E, Morelli G, Russell JE, Urwin R, et al. Multilocus sequence typing: A portable approach to the identification of clones within populations of pathogenic microorganisms. Proc Natl Acad Sci U S A 1998;95:3140–5. https://doi.org/10.1073/pnas.95.6.3140.

[97]	Harmsen D, Claus H, Witte W, Rothgänger J, Claus H, Turnwald D, et al. Typing of Methicillin-Resistant Staphylococcus aureus in a University Hospital Setting by Using Novel Software for spa Repeat Determination and Database Management. J Clin Microbiol 2003;41:5442–8. https://doi.org/10.1128/JCM.41.12.5442-5448.2003.

[98]	Yamamoto S, Kasai H, Arnold DL, Jackson RW, Vivian A, Harayama S. Phylogeny of the genus Pseudomonas: Intrageneric structure reconstructed from the nucleotide sequences of gyrB and rpoD genes. Microbiology 2000;146:2385–94. https://doi.org/10.1099/00221287-146-10-2385.

[99]	Peeters K, Willems A. The gyrB gene is a useful phylogenetic marker for exploring the diversity of Flavobacterium strains isolated from terrestrial and aquatic habitats in Antarctica. FEMS Microbiol Lett 2011;321:130–40. https://doi.org/10.1111/j.1574-6968.2011.02326.x.

[100]	Janda JM, Abbott SL. 16S rRNA gene sequencing for bacterial identification in the diagnostic laboratory: Pluses, perils, and pitfalls. J Clin Microbiol 2007;45:2761–4. https://doi.org/10.1128/JCM.01228-07.

[101]	Fisher MM, Triplett EW. Automated approach for ribosomal intergenic spacer analysis of microbial diversity and its application to freshwater bacterial communities. Appl Environ Microbiol 1999;65:4630–6. https://doi.org/10.1128/aem.65.10.4630-4636.1999.

[102]	Stoppani N, Colussi S, Pastorino P, Prearo M, Sciuto S, Altinok I, et al. 16S-23S rRNA Internal Transcribed Spacer Region (ITS) Sequencing: A Potential Molecular Diagnostic Tool for Differentiating Lactococcus garvieae and Lactococcus petauri. Microorganisms 2023;11:1320. https://doi.org/10.3390/microorganisms11051320.

[103]	Mollet C, Drancourt M, Raoult D. rpoB sequence analysis as a novel basis for bacterial identification. Mol Microbiol 1997;26:1005–11. https://doi.org/10.1046/j.1365-2958.1997.6382009.x.

[104]	Adékambi T, Shinnick TM, Raoult D, Drancourt M. Complete rpoB gene sequencing as a suitable supplement to DNA-DNA hybridization for bacterial species and genus delineation. Int J Syst Evol Microbiol 2008;58:1807–14. https://doi.org/10.1099/ijs.0.65440-0.
